# Estrogen-induced FXR1 promotes endocrine resistance and bone metastasis in breast cancer via BCL2 and GPX4

**DOI:** 10.3389/fcell.2025.1563353

**Published:** 2025-03-24

**Authors:** Yinzhong Shang, Tingfang Cao, Xin Ma, Le Huang, Mingming Wu, Junchao Xu, Jiarui Wang, Hao Wang, Sheng Wu, Vijay Pandey, Zhengsheng Wu, Weijie Zhang, Peter E. Lobie, Xinghua Han, Tao Zhu

**Affiliations:** ^1^ Department of Oncology, Division of Life Sciences and Medicine, The First Affiliated Hospital of USTC, Center for Advanced Interdisciplinary Science and Biomedicine of IHM, National Key Laboratory of Immune Response and Immunotherapy, University of Science and Technology of China, Hefei, China; ^2^ Shenzhen Bay Laboratory, Institute of Biomedical Health Technology and Engineering, Shenzhen, China; ^3^ Tsinghua Shenzhen International Graduate School, Institute of Biopharmaceutical and Health Engineering, Shenzhen, China; ^4^ Department of Pathology, The First Affiliated Hospital of Anhui Medical University, Hefei, China; ^5^ Zhejiang Provincial Key Laboratory of Cancer Molecular Cell Biology, Life Sciences Institute, Zhejiang University, Hangzhou, China; ^6^ Department of Orthopaedic Surgery, The Second Affiliated Hospital, School of Medicine, Zhejiang University, Hangzhou, China; ^7^ Anhui Key Laboratory of Molecular Oncology, Hefei, China

**Keywords:** estrogen, FXR1, apoptosis, ferroptosis, anti-estrogen resistance

## Abstract

Estrogen signaling dysregulation plays a critical role in the development of anti-estrogen resistance and bone metastasis of ER+ mammary carcinoma. Using quantitative proteomic screening, we identified FXR1 as an estrogen-regulated RNA-binding protein associated with anti-estrogen resistance. Mechanistically, estrogen and IGF1 facilitate FXR1 protein translation via the PI3K/AKT/mTOR/EIF4E pathway. FXR1 enhances cellular resistance to apoptosis and ferroptosis by facilitating the maturation of BCL2 pre-mRNA and stabilizing GPX4 mRNA, respectively. Anti-estrogen resistant cells exhibit elevated FXR1 expression, and FXR1 depletion restores their sensitivity to tamoxifen. Moreover, combining FXR1 depletion with a ferroptosis inducer induces synergistic lethal in anti-estrogen resistant cells. Finally, we provide proof-of-concept evidence supporting FXR1 antagonism as a potential treatment for bone metastases in ER+ breast cancer. Our findings highlight FXR1 as a promising therapeutic target to improve existing therapeutic regimes for ER+ breast cancer patients.

## 1 Introduction

mRNA-binding proteins (mRBPs) are a group of proteins that are capable of interacting with messenger RNAs. RBPs have been implicated as important regulators in various physiologic and pathologic conditions by regulating gene expression post-transcriptionally, including RNA splicing, modification, localization, stability, and protein translation (Gerstberger et al., 2014). In cancer, aberrations in RBP expression facilitate cell survival, metastasis and therapeutic resistance, resulting in progression (Kang et al., 2020). Although over 1,500 RBPs have been identified in the entire human genome using recent high-throughput screening, a paucity of RBPs have been functionally delineated (Mohibi et al., 2019).

Breast cancer ranks as the most prevalent cancer among women globally, with over 70% of patients falling into the estrogen receptor-positive (ER+) category (Waks and Winer, 2019). Anti-estrogen therapy has significantly improved the survival outcomes for ER+ breast cancer patients; however, the emergence of therapy resistance remains a challenge for durability of treatment (Barone et al., 2022). The disease in anti-estrogen resistant patients at advanced stages are often accompanied by metastasis, especially bone metastasis (Hess et al., 2006). The majority of primary ER+ breast cancer and approximately 30%–50% of recurrent ER+ breast cancer are hormone-dependent and rely on ERα signaling (Miziak et al., 2023); therefore, both types of patients benefit clinically from treatments with fulvestrant or aromatase inhibitors (Ferreira Almeida et al., 2020). Tremendous efforts have been undertaken to identify mechanisms driving sustained ER signaling in anti-estrogen resistant cells by identifying the aberrantly expressed estrogen responsive genes. However, almost all the previous studies of have been exclusively based on the transcriptional profiling of estrogen stimulation. Given the substantial difference between the transcriptional regulation and translational regulation of gene expression, we performed quantitative proteomic analysis to screen for estrogen responsive mRBPs. We identified FXR1 as a potential therapeutic target in ER+ breast cancer.

## 2 Materials and methods

### 2.1 Cell lines and reagents

MCF-7, T47D, BT474, MDA-MB-231, and HEK-293 T cell lines used in this study were purchased from the American Type Culture Collection (ATCC). All cells were cultured under standard conditions (37°C, 5% CO2) in a culture medium with 1% penicillin/streptomycin and 10% fetal bovine serum (Gibco). MCF-7 TAMR and T47D TAMR cell lines were derived from parental cells maintained in culture with 1 μM tamoxifen (TargetMol, United States; T6906) for at least 6 months. MCF-7 LTED cells were cultured in phenol red-free RPMI-1640 (Gibco) medium containing 5% estrogen-deprived serum for at least 6 months. All cell lines tested negative for *mycoplasma*. The sources of all the reagents are described in Supplementary Table S1.

### 2.2 Plasmids and established stable cells

PCR-amplified BCL2, GPX4, and ESR1 were cloned into the pSin vector to generate expression plasmids. FXR1, FXR1-KH, and FXR1-RGG were subcloned into the pSin-3×Flag vector. R-FXR1 contains synonymous mutations of C118A, C120G, G123A, T126C, A129T, T132C, T135C, and A138G. The 3′UTR of GPX4 was cloned into the psiCHECK-2 vector (Promega). GPX4 3′UTR mutations were generated using the QuickChange site-directed mutagenesis kit (Agilent Technologies). All shRNA plasmids directed to human genes were obtained from The RNAi Consortium (MISSION® TRC shRNA library, Sigma-Aldrich). The sgRNAs targeting FXR1 were inserted into the multiple cloning sites of the lentiCRISPRv2 vector. Sequences of shRNAs, sgRNAs, and primers are listed in Supplementary Table S1. The pSin-FXR1 and shRNA viral particles were generated by co-transfection of the constructs with pMD2. G and psPAX2 into HEK-293T cells using calcium phosphate. Viral particles were collected 24 and 48 h after transfection and filtered through a 0.45 μm filter unit (Millipore). Cells were infected with polybrene (8 μg/mL, Sigma-Aldrich) for 48 h. Subsequently, stably integrated cells were selected with 2 μg/mL puromycin for 1 week.

### 2.3 RT-qPCR and western blot

Total RNA was extracted from cells or RIP samples using Trizol (Takara) according to the instructions. Real-time quantitative PCR was performed using the TransScript® Top/Tip Green qRT-PCR SuperMix (Transgen). Cells were lysed with RIPA buffer (Beyotime) supplemented with a protease inhibitor cocktail (Targetmol), and the protein concentration was determined using the BCA Protein Assay Kit (Abcam). Western blot images were collected and processed using the ImageQuant LAS4000 Mini (GE Health). Primer sequences and antibody information are listed in Supplementary Table S1.

### 2.4 Cell viability and cell death assays

For cell viability assays, cells were seeded in 96-well plates. After treatment with the drugs specified, the culture medium was replaced with fresh medium supplemented with 10% MTT. After incubating for 2 h at 37°C, the absorbance at OD570 was measured for each well. In the case of cell death assays, cells were seeded in a 6-well plate 24 h before treatment. Harvested cells were then resuspended in 500 µL PBS buffer containing 2 μg/mL propidium iodide and stained for 15 min. The percentage of dead cells was subsequently analyzed using a flow cytometer (BD LSRFortessa).

### 2.5 Cell apoptosis

The Dead Cell Apoptosis Kit with Annexin V FITC & Propidium Iodide (Invitrogen) was used. Briefly, cells from a 6 cm culture dish were harvested using trypsin without EDTA, washed with cold PBS, and resuspended in 200 µL of 1× annexin-binding buffer containing 5 µL of FITC Annexin V and 1 µL of the 100 μg/mL PI working solution. The cells were then incubated at room temperature for 15 min and subsequently analyzed by flow cytometry (BD LSRFortessa) for cell apoptosis assessment.

### 2.6 Isobaric tags for relative and absolute quantitation (iTRAQ)

MCF-7 cells were cultured in medium containing 5% estrogen-deprived serum for 6 days, followed by a 48-hour treatment with estrogen or DMSO. Protein labeling was conducted in a single tube according to the instructions provided in the iTRAQ Reagent-8plex Multiplex Kit. The labeled samples (DMSO-1 = iTRAQ 115; DMSO-2 = iTRAQ 116; DMSO-3 = iTRAQ 117; E2-1 = iTRAQ 118; E2-2 = iTRAQ 119; E2-3 = iTRAQ 121) were combined into a single sample mixture for Liquid Chromatography-Mass Spectrometry analysis. The iQuant software was utilized to analyze the labeled peptides with isobaric tags quantitatively, and the iTRAQ data analyses were performed by BGI Genomics, Shenzhen, China (Wen et al., 2014).

### 2.7 Polysome fractionation by sucrose gradients

As described (George et al., 2021), the cells were treated with 0.1 mg/mL cycloheximide for 15 min. The collected cell lysate was loaded onto a sucrose gradient column (5%–50% w/v) and centrifuged at 38,000 rpm for 3 h at 4°C. Fractions were collected using the density gradient fractionation system Piston Gradient Fractionator (BIOCOMP). Total RNA was extracted from the fractions and analyzed by RT-qPCR.

### 2.8 mRNA stability assay

As described (Yuan et al., 2022) cells were treated with 5 μM actinomycin D for specified time points. Total RNA extracted from the cells was utilized to generate a cDNA library through reverse transcription. Subsequently, real-time quantitative PCR was employed to measure the expression levels of GPX4 mRNA.

### 2.9 RNA-IP and RIP-seq

The cells were lysed and immunoprecipitated using FXR1 antibody, with mouse IgG as a negative control. The mRNA that FXR1 or IgG precipitated was reverse transcribed into cDNA and analyzed through RT-qPCR. For the RIP-seq, the purified RNA underwent reverse transcription PCR, end repair, adapter ligation, PCR enrichment, and deep sequencing. RIP samples were normalized using INPUT samples to identify highly enriched regions. RIP-seq data analyses were assisted by DIATRE Biotechnology in Shanghai, China.

### 2.10 Dual-luciferase reporter assay

HEK-293T cells were co-transfected with pSin-FXR1 and psiCHECK-2 Vector plasmid containing either the GPX4-3’UTR or a 3’UTR mutation mRNA fragment. 48 h post-transfection, cells were harvested, and 100 μL lysis buffer was added to each sample. Subsequently, the sample supernatant was utilized to measure luciferase activity with Firefly luciferase activity normalized to Renilla luciferase activity (Beyotime).

### 2.11 Lipid ROS assay

The dispersed cells were incubated with a serum-free medium containing 10 μM C11 BODIPY 581/591 at 37°C for 30 min to label lipid reactive oxygen species (ROS). The levels of lipid ROS were analyzed using flow cytometry (BD LSRFortessa).

### 2.12 GSH/GSSG analysis

Cells were plated in 6-well plates and harvested when reaching 90% confluency. The levels of reduced glutathione (GSH) and oxidized glutathione (GSSG) were determined using a GSH/GSSG detection kit (Beyotime S0053). The GSH and GSSG levels in each sample were normalized to the corresponding protein concentration.

### 2.13 Transmission electron microscopy

FXR1 depleted or control cells were fixed in 2.5% glutaraldehyde for 12 h at 4°C, then fixated in 2% OsO4 for 2 h at 4°C. Subsequently, the samples underwent dehydration using an ethanol gradient and were embedded in Eponate 12 resin. Ultrathin sections were prepared and counterstained with uranyl acetate and lead citrate. The images were obtained using a transmission electron microscope (120 kV; Tecnai G2 Spirit, FEI).

### 2.14 Xenograft mouse model

Animal studies were approved by the Animal Research Ethics Committee of the University of Science and Technology of China (Ethics number: USTCACUC212201042). The 5-week-old female BALB/c nude mice were purchased from SLAC laboratory animals (Shanghai, China). For ER+ breast cancer cells, mice were implanted with a 60-day-release 17β-estradiol pellet (0.36 mg, Innovative Research of America). Subsequently, 2 × 10^6^ MCF-7 cells stably expressing shFXR1 or control were injected into the mammary fat pads of the mice. After the xenograft reached 150 mm^3^, mice were treated with tamoxifen (60-day-release tamoxifen pellet 5 mg, Innovative Research of America) and IKE (25 mg/kg every 2 days via *i. p*). Xenograft volumes (length (mm) × (width (mm))^2^/2) were measured every 3 days using a digital caliper.

The bone metastasis model was established as reported (Kuchimaru et al., 2018). 1.5 × 10^6^ luciferase-labeled MCF-7 TAMR cells were injected into the caudal arteries of mice. After 2 weeks, distinct bone metastasis signals were observed, and the mice were randomly divided into four groups: vehicle, fulvestrant (2 mg, every 2 days via *s. c*), IKE (25 mg/kg, every 2 days via *i. p*), and fulvestrant + IKE. Mice were injected with 150 μg/g of D-luciferin (12 mg/mL in PBS) and imaged using a PerkinElmer IVIS Spectrum system. The bioluminescent imaging was quantified using Living Image 4.5 software. Micro-CT scans were performed for the dissected hind legs of euthanized mice using NMC-200(NEMO, China).

### 2.15 Tissue microarray and immunohistochemistry (IHC)

The tissue microarray (ZL-BrcSur180) was obtained from Zhuoli Biotechnology Co., Ltd. (Shanghai, China), and detailed patient information is listed in Supplementary Table S2. The clinical research protocol was approved by the Biomedical Ethics Committee of Zhuoli Biotech (No. LLS M-15-1). The study is compliant with all relevant ethical regulations. Paraffin sections from mouse xenografts were dewaxed in xylene and rehydrated in graded alcohol. After the inactivation of endogenous peroxidase using 3% hydrogen peroxide, high-pressure heat repair was performed in citrate buffer. Sections were preincubated in 5% BSA for 30 min to prevent nonspecific staining, followed by incubation with antibodies at 4°C overnight. DAB chromogenic and hematoxylin staining were performed after the secondary antibody incubation.

### 2.16 Statistical analysis

The Kaplan–Meier plotter database was used to analyze the effect of genes on cancer patient survival. Data are expressed as mean ± standard deviation (s.d.) from three independent experiments. Statistical analyses were conducted using GraphPad Prism nine or R software (version 4.4.1). Two-tailed unpaired Student’s t-test and one- or two-way analysis of variance (ANOVA) were used for group comparisons, and a significance level of p < 0.05 was considered statistically significant.

## 3 Results

### 3.1 Screening of estrogen-regulated mRBPs in ER+ breast cancer

**FIGURE 1:**
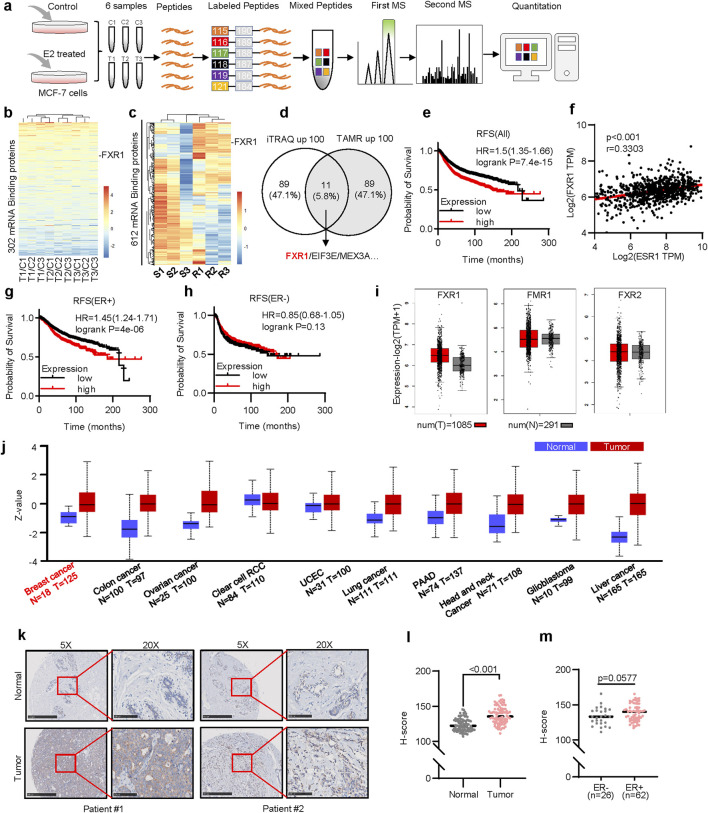
Screening of estrogen-regulated mRBPs in ER+ breast cancer. **(a)**, The schematic of iTRAQ quantitative proteomics. MCF-7 cells were treated with 10 nM estrogen for 48 h after 6 days of estrogen deprivation. **(b)**, Heat map representation of the 302 mRBP gene ratios (Treat/Control) in the iTRAQ quantitative proteomics. Each group represents three independent experiments. **(c)**, The heatmap plotted the relative expression levels of 612 mRBPs in tamoxifen-resistant(R) and parental(S) MCF-7 cells. **(d)**, Schematic diagram of 11 potential mRBPs in breast cancer. The left circle shows 100 proteins upregulated in quantitative proteomics. The right circle shows 100 genes upregulated in tamoxifen-resistant cells. **(e)**, Kaplan–Meier plots of RFS in breast cancer patients with different levels of FXR1 expression. **(f)**, Correlation analysis of FXR1 and ESR1 in TCGA ER+ breast cancer samples by GEPIA 2. **(g, h)**, Kaplan–Meier plots of RFS in ER+ **(g)** and ER- **(h)** patients with different levels of FXR1 expression. **(i),** Expression levels of FXR1, FMR1, and FXR2 in normal tissue (n = 291) and tumor tissue (n = 1,085) were analyzed by GEPIA 2. **(j)**, Protein levels of FXR1 in different cancers (tumor and normal samples). **(k)**, IHC staining of FXR1 of the representative patients in breast cancer tissue microarray. Scale bar: 500 μm (5✕), 100 μm (20✕). **(l)**, IHC H-scores of FXR1 in breast normal and tumor sections. **(m)**, IHC H-scores of FXR1 in ER+ and ER- breast tumor sections. Results are shown as mean ± S.D. *P < 0.05; **P < 0.01; ***P < 0.001; ns not significant (Unpaired two-tailed Student’s t test).

To identify the possible mRBPs that are modulated by estrogen, a quantitative proteomic analysis utilizing iTRAQ was conducted by use of MCF-7 cells in the presence or absence of estrogen (17-beta estradiol) as indicated (Figure 1A). A total of 896,911 spectrums were generated; 54,468 peptides and 7,462 proteins were identified with 1% False Discovery Rate (FDR). Proteins exhibiting altered expression greater than 1.2-fold with a p-value less than 0.05 were classified as differentially expressed. 511 proteins manifested significantly altered expression levels between DMSO and E2-treated groups. Gene Ontology (GO) revealed that differentially expressed proteins were primarily enriched in cellular process, metabolic process, and biological regulation (Supplementary Figure S1A). Further analysis suggested that the significantly altered genes were enriched in sphingolipid metabolism, hematopoietic cell lineage, and estrogen signaling pathway (Supplementary Figure S1B).

Among the 692 known mRBPs (Gerstberger et al., 2014), 302 were identified in our quantitative proteomics analysis (Figure 1B). Further, expression profiling of 612 mRBP genes in tamoxifen-resistant cells and cognate control cells were obtained from the GSE164529 dataset, excluding 80 genes not recorded (Figure 1C) (Jones et al., 2021). Cross-analysis of the top 100 upregulated mRBPs in response to estrogen treatment and the top 100 upregulated mRBPs in tamoxifen-resistant cells identified 11 overlapping mRBP genes in both datasets (Figure 1D). To assess the potential clinical implication of these 11 genes in breast cancer, their clinical prognoses were further analyzed. Higher expression of FXR1, EIF3E, and MEX3A (Figure 1E; Supplementary Figure S1C, D) but not the remaining eight genes (Supplementary Figure S1E–L) was subsequently identified to be associated with worse recurrence-free survival (RFS). The correlation analysis conducted using the ER+ breast cancer TCGA dataset indicates that only FXR1 shows a positive correlation with ESR1, while EIF3E and MEX3A have no correlation with ESR1 expression levels (Figure 1F; Supplementary Figure S1L, M) (Li et al., 2021). Notably, higher expression of FXR1 was significantly associated with a worse RFS (Figures 1G, H), overall survival (OS) (Supplementary Figure S1O, P), and distant metastasis-free survival (DMFS) (Supplementary Figure S1Q, R) exclusively in ER+ but not in ER- cohorts.

FXR1, FMR1, and FXR2 belong to the fragile X messenger ribonucleoprotein family (Li et al., 2021), whereas only FXR1 was increased in expression in breast cancer tissues compared to normal tissues (Figure 1I). Pan-cancer analysis revealed elevated FXR1 protein levels across several tumors, including breast, liver, and lung cancers (Figure 1J) (Bartha and Győrffy, 2021; Chandrashekar et al., 2022). Tissue microarrays, including 90 normal breast tissue and 90 breast cancer samples (Supplementary Figure S1S), were used to assess FXR1 expression. The patient information is shown in Supplementary Table S2. Immunohistochemistry analysis suggested higher FXR1 expression in cancer tissues (Figures 1K, L). In addition, a tendency of elevated expression of FXR1 was observed in ER+ compared to ER- breast cancers (Figure 1M). These findings suggest that FXR1 is potentially oncogenic in ER+ breast cancer.

### 3.2 ER signaling induces FXR1 translation via eIF4E and eIF4EBP1

**FIGURE 2:**
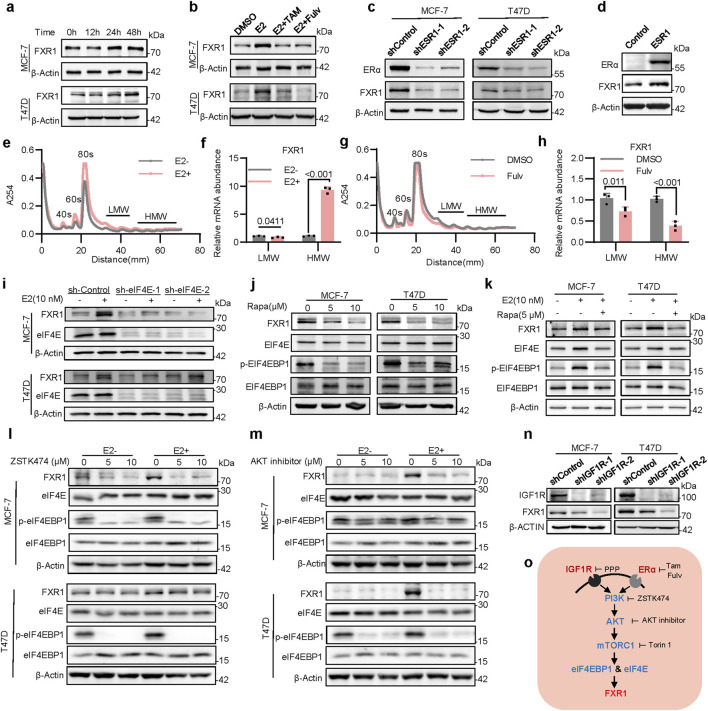
Estrogen induces FXR1 translation via eIF4E and eIF4EBP1. **(a)**, Immunoblot assessment of FXR1 levels. Cells were treated with 10 nM estrogen for 0h, 12h, 24h and 48h. **(b)**, Immunoblot assessment of FXR1 levels. Cells were treated with estrogen (10 nM) in combination with tamoxifen (1 μM) and fulvestrant (1 μM) for 48 h. **(c)**, Immunoblot assessment of FXR1 and ERα levels in ESR1 depleted MCF-7 and T47D cells. **(d)**, Immunoblot assessment of FXR1 and ERα levels in MDA-MB-231 cells with forced expression of ESR1 and control cells. **(e)**, Representative polysome traces of MCF-7 cells in estrogen-deprived and stimulated (10 nM) conditions. Distance (mm) 10-30: mRNA ribonucleo protein (mRNP)/monosome; distance (mm) 30-50: light polysome (LMW); distance (mm) 50-70: heavy polysome (HMW). **(f)**, Relative abundance of FXR1 mRNA in LMW or HMW shown in **(e)**. **(g)**, Representative polysome traces of MCF-7 cells after DMSO or fulvestrant (1 μM) treatment 48h. **(h)**, Relative abundance of FXR1 mRNA in LMW or HMW shown in **(g)**. **(i)**, Immunoblot assessment of FXR1 and eIF4E levels. MCF-7 and T47D sh-Control or sh-eIF4E cell lines were treated with estrogen (10 nM) for 48 h. **(j, k)**, Immunoblot assessment of FXR1, eIF4E, eIF4EBP1 and phosphorylated eIF4EBP1 levels. MCF-7 and T47D cells were treated with different concentrations of rapamycin **(j)** or combined with estrogen **(k)**. **(l, m)**, Immunoblot assessment of FXR1, eIF4E, eIF4EBP1 and phosphorylated eIF4EBP1 protein levels. In the presence or absence of estrogen, MCF-7, and T47D cells were treated with different concentrations of ZSTK474 **(l)** or AKT inhibitors **(m)**. **(n)**, Immunoblot assessment of FXR1 and IGF1R levels in IGF1R depleted MCF-7, T47D, and control cells. **(o)**, Schematic diagram of the pathway that estrogen and IGF1 regulate FXR1 translation. Results are shown as mean ± S.D. *P < 0.05; **P < 0.01; ***P < 0.001; ns not significant (Unpaired two-tailed Student’s t test).

To determine the mechanism by which estrogen promotes FXR1 expression, MCF-7 and T47D cells were exposed to estrogen for the indicated periods, and markedly escalated FXR1 levels were observed at 24- and 48-h post-exposure (Figure 2A), whereas FXR1 mRNA levels remained unchanged. The TFF1 level was examined as a positive control (Supplementary Figures S2A, B). It was also observed that estrogen increased FXR1 expression in a dose-dependent manner (Supplementary Figure S2C). The estrogen-induced level and endogenous level of FXR1 were readily abrogated by tamoxifen or fulvestrant (Figure 2B; Supplementary Figure S2D), indicating that estrogen induced FXR1 expression via ERα. Consistently, ESR1 depletion in ER+ breast cancer cells resulted in reduced FXR1 expression (Figure 2C). In contrast, forced expression of ESR1 in ER- MDA-MB-231 cells significantly increased FXR1 levels (Figure 2D). As the data suggested that estrogen regulates FXR1 expression at the post-transcriptional level, we further examined if estrogen could affect FXR1 expression at the translational level. Using ribosome density gradient centrifugation to separate polysomes from cells under different culture conditions (Figure 2E), it was observed that estrogen significantly increased the level of FXR1 mRNA in the heavy polysomes (Figure 2F). In contrast, fulvestrant treatment significantly decreased the FXR1 mRNA content in the heavy polysomes (Figures 2G,H). The data suggests that estrogen potentially promotes the translation of FXR1.

To determine the eukaryotic translation initiation factors (eIFs) that mediate estrogen-induced FXR1 expression, the correlation of multiple eIFs with ESR1 in the TCGA database was performed (Supplementary Figure S2E). It was observed that the level of eIF4E was positively correlated with that of ESR1 (Supplementary Figure S2F). In contrast, the level of eIF4EBP1 was negatively correlated with ESR1 (Supplementary Figure S2G). The expression level of eIF4E was higher in luminal breast cancer than that in triple-negative breast cancer (Supplementary Figure S1H), whereas the expression level of eIF4EBP1 showed the opposite tendency (Supplementary Figure S2I). eIF4EBP1 was known to inhibit the translation initiation activity of eIF4E by interaction, whereas eIF4EBP1 phosphorylation releases this inhibition (Martineau et al., 2013). To determine if estrogen regulates FXR1 expression via the signaling of eIF4EBP1 and eIF4E, the possible regulation of FXR1 expression by eIF4E was examined. As eIF4E depletion using shRNA significantly downregulated FXR1 expression (Supplementary Figure S2J, K), estrogen-induced FXR1 expression was significantly abrogated by eIF4E depletion (Figure 2I). To validate further that eIF4E is involved in the translational regulation of FXR1, rapamycin, an mTOR specific inhibitor was applied to inhibit the translation activity of eIF4E; and it was subsequently observed that FXR1 expression was significantly diminished (Figure 2J). Consistently in the presence of rapamycin, estrogen-induced phosphorylation of eIF4EBP1 and increased FXR1 expression were abrogated (Figure 2K). As eIF4EBP1 is known to be regulated by the PI3K/AKT/mTORC1 signaling pathway (Popova and Jücker, 2021), the cells were subsequently treated with inhibitors of PI3K, AKT, and mTORC1 to determine if this signaling pathway is involved in the translational regulation of FXR1. As expected, blockage of this signaling pathway abolished estrogen-induced FXR1 expression (Figures 2L, M; Supplementary Figure S2L).

Interestingly, analysis of CPTAC databases indicates that the protein level of FXR1 is significantly elevated in breast cancer samples with altered signaling pathways of mTOR and MYC but not NRF2, WNT, or HIPPO (Supplementary Figure S2M) (Chandrashekar et al., 2022; Zhang et al., 2022; Chen et al., 2023). It is well-known that PI3K/AKT/mTORC1 signaling plays a key role in the cross-talk between ERα and the receptor tyrosine kinases such as IGF1R in ER+ breast cancer (Massarweh et al., 2008; Ianza et al., 2021). It was therefore examined whether IGF1R signaling could regulate FXR1 expression. IGF1R depletion in ER+ breast cancer cells significantly reduced FXR1 expression (Figure 2N), whereas activation of IGF1R signaling by IGF1 led to increased FXR1 protein expression (Supplementary Figure S2N). Antagonism of IGF1R signaling by the IGF1R specific inhibitor picropodophyllin (PPP) reduced FXR1 protein expression (Supplementary Figure S2O). Consistently, phosphorylated levels of eIF4EBP1 increased following IGF1R activation but decreased as a result of IGF1R inhibition (Supplementary Figures S2N, O). Thus, these findings indicate that both ERα signaling and IGF1R signaling promote FXR1 translation via the PI3K-AKT-mTORC1-eIF4E signal pathway (Figure 2O).

### 3.3 FXR1 is oncogenic in breast cancer

**FIGURE 3:**
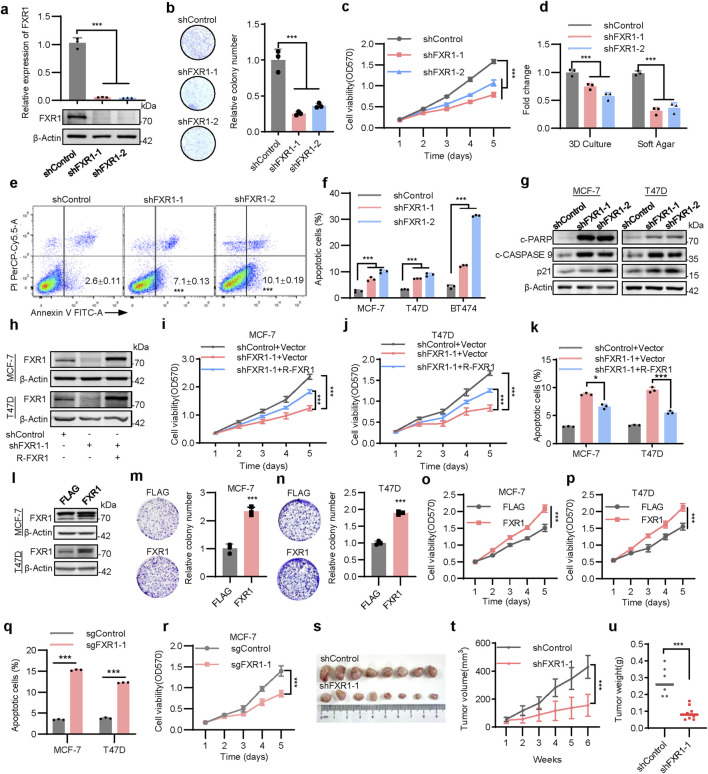
FXR1 enhances oncogenicity of breast cancer cells. **(a)**, qPCR (n = 3 biological replicates) and immunoblot analysis of FXR1 expression in FXR1 depleted MCF-7 and control cells. **(b)**, Foci formation assay was performed in FXR1 depleted MCF-7 and control cells. Representative images (left) and statistical analyses (right) of the colonies were shown. **(c)**, MTT assay showing relative cell viability in FXR1 depleted MCF-7 and control cells. **(d)**, Statistical analyses of the FXR1 depleted MCF-7 cell colonies in 3D culture and soft agar colony formation assays. **(e)**, Early apoptotic population (FITC+/PerCP-Cy5.5-) in FXR1 depleted MCF-7 cells was determined by flow cytometry. **(f)**, Statistical analyses of the apoptotic FXR1 depleted MCF-7, T47D, and BT474 cells by flow cytometry. **(g)**, Immunoblot assessment of apoptosis-associated marker levels in FXR1 depleted MCF-7, T47D, and control cells. **(h–k)**, FXR1 depleted MCF-7 and T47D cells rescued with empty vector or R-FXR1 plasmid**.** Immunoblot assessment of FXR1 levels **(h)**, MTT assay showing relative cell viability **(i, j)**, and cell apoptosis determined by flow cytometry **(k)**. **(l)**, Immunoblot assessment of FXR1 levels after forced expression of FXR1 MCF-7 and T47D cells. **(m, n)**, Foci formation assay was performed in MCF-7 (**m**) and T47D **(n)** cells with forced expression of FXR1. **(o, p)**, MTT assay showing relative cell viability in MCF-7 **(o)** and T47D **(p)** cells with forced expression of FXR1. **(q)**, Early apoptotic population in FXR1-deleted MCF-7 and T47D cells was determined by flow cytometry. **(r)**, MTT assay showing relative cell viability in FXR1-deletion MCF-7 and control cells. **(s–u)**, MCF-7 cells stably expressing pLKO1 or shFXR1 plasmid were injected into nude mice (n = 8/group). Tumor size was measured starting at 7 days after injection. The tumor picture **(s)**, tumor growth curves **(t)**, and tumor weight **(u**) was shown. Results are shown as mean ± S.D. *P < 0.05; **P < 0.01; ***P < 0.001; ns not significant (Unpaired two-tailed Student’s t test in **(m, n, q, u)**, one-way ANOVA test in **(a, b, d, f, k)** others two-way ANOVA test.).

The functional roles of FXR1 in breast cancer were further addressed. FXR1 depletion by shRNA significantly impaired the viability and foci formation of MCF-7 (Figures 3A–C), T47D, and BT474 cells (Supplementary Figures S3A–F). FXR1 depletion also impaired anchorage-independent growth, as revealed by soft agar assays and repressed cell growth in 3D Matrigel (Figure 3D; Supplementary Figures 3G, H). Flow cytometry analysis showed that FXR1 depletion resulted in cell cycle arrest with an increased proportion of cells in the G0/G1 phase (Supplementary Figures S3I–L). Analysis of apoptotic cells using Annexin V-FITC staining followed by flow cytometry revealed that FXR1 depletion increased the percentage of apoptotic cells in ER+ breast cancer cells (Figures 3E, F; Supplementary Figures S3M). Consistently, FXR1 depletion upregulated the expression of p21, cleaved-CASPASE 9 and cleaved- PARP (Figure 3G).

To prevent possible off-target effects of FXR1 shRNA, shRNA-resistant FXR1 (R-FXR1) was introduced into FXR1-depleted cells, which partially restored the reduced cell viability observed after FXR1 depletion (Figures 3H–J) and reversed FXR1 depletion promoted apoptosis (Figure 3K). Moreover, forced expression of FXR1 in MCF-7 and T47D cells significantly increased foci formation and cell viability (Figures 3L–P). FXR1 deletion using the CRISPR-Cas9 system confirmed that FXR1 deletion led to increased cell apoptosis and reduced cell viability (Figures 3Q,R; Supplementary Figures S3N–P). In line with *in vitro* findings, FXR1 depletion substantially diminished the growth of tumor xenografts in nude mice (Figures 3S–U). Taken together, these results indicate that FXR1 is oncogenic in breast cancer.

### 3.4 FXR1 regulates apoptosis via BCL2

**FIGURE 4:**
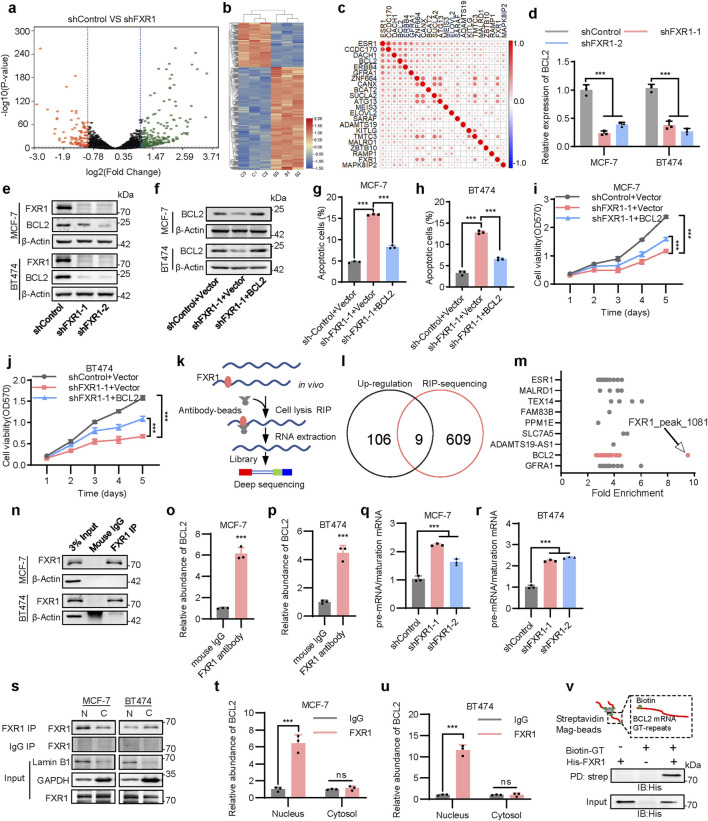
FXR1 regulates apoptosis by promoting BCL2 mRNA maturation. **(a)**, Volcano map showing all gene expression changes in MCF-7 cells expressing shControl or shFXR1 plasmids. **(b)**, Differential gene clustering diagram. Red indicates highly expressed genes, and blue indicates low expressed genes. **(c)**, Heat map plots the Pearson correlation coefficient between candidate genes and ESR1 from breast cancer TCGA data. **(d)**, qPCR analysis of BCL2 expression in FXR1 depleted cells. **(e)**, Immunoblot assessment of FXR1 and BCL2 expression levels in FXR1 depleted cells. **(f)**, Immunoblot assessment of BCL2 expression level in FXR1 depleted cells rescued with forced expression of BCL2. **(g, h)**, Early apoptotic (FITC+/PerCP-Cy5.5-) population in FXR1 depleted MCF-7 **(g)** and BT474 **(h)** cells rescued with forced expression of BCL2. **(i, j)**, MTT assay showing relative cell viability in FXR1 depleted MCF-7 **(i)** and BT474 **(j)** cells rescued with forced expression of BCL2. **(k)**, A schematic of RNA immunoprecipitation experiment for identifying genes associated with FXR1. **(l)**, Venn diagram of RNA-seq and RIP–seq showing that the mRNA of nine genes was bound to and upregulated by FXR1. **(m)**, Fold enrichment of FXR1 binding peaks among nine genes in RIP experiment. **(n)**, Immunoblot assessment of enriched FXR1 in RIP experiments performed in the cells. **(o, p)**, qPCR analyzed the association of FXR1 with BCL2 mRNA by RIP assays in MCF-7 **(o)** and BT474 **(p)** cells. **(q, r)**, qPCR analyzed the ratio between pre-mRNA and mature mRNA of BCL2 in FXR1 depleted MCF-7 **(q)** and BT474 **(r)** cells. **(s)**, Immunoblot assessment of enriched FXR1 in RIP experiments performed in cytoplasmic and nuclear fractions of the cells. **(t, u)**, qPCR analyzed the association of FXR1 with BCL2 by RIP assays in cytoplasmic and nuclear fractions of MCF-7 **(t)** and BT474 **(u)** cells. **(v)**, Purified recombinant His–FXR1 (10 μg) was incubated with biotin–GT repeat (200 nM) for 4 h. Pull-down assays were performed with streptavidin agarose beads. Results are shown as mean ± S.D. *P < 0.05; **P < 0.01; ***P < 0.001; ns not significant (Unpaired two-tailed Student’s t test in **(o, p, t, u)**, two-way ANOVA test in **(i, j)** others one-way ANOVA test).

To gain mechanistic insight into the functions of FXR1 in breast cancer, RNA-seq analysis of MCF-7 cells with FXR1 depletion was performed. An average of 4.8 million uniquely mapped reads were yielded, uncovering 12,552 distinct transcripts (FPKM>1). Further analysis highlighted 265 genes with increased expression and 115 genes with decreased expression in FXR1-depleted cells compared to controls (fold change> 2, p < 0.05) (Figures 4A, B). GO analysis indicated pronounced enrichment of genes involved in membrane components, transmembrane transport, and apoptotic processes (Supplementary Figure S4A). Given the capacity of FXR1 to propel cancer progression, the diminished expression of several candidate oncogenic genes due to FXR1 depletion, including STMN3, NELL2, KITLG, and PCDH19, were examined in ER+ breast cancer cells (Supplementary Figures S4B–E). Again, KEGG pathway analysis linked the differentially expressed genes primarily to the estrogen signaling pathway and endocrine resistance (Supplementary Figures S4F).

To verify that FXR1 is involved in regulating estrogen signaling, we analyzed the relationship between FXR1-regulated genes and ESR1. TCGA data analysis showed that multiple FXR1-regulated genes were positively correlated with ESR1 and were highly expressed in ER+ BC patients (Figure 4C; Supplementary Figures S4G). As suggested by the RNA-seq data, RT-qPCR analysis was performed to verify that loss of FXR1 resulted in significant downregulation of several estrogen-responsive genes including BCL2, ATG13, and ELOVL2 (Supplementary Figures S4H) (Dong et al., 1999; González-Bengtsson et al., 2016; Zhou et al., 2021). The above results indicate that FXR1 may participate in the regulation of genes involved in ER signaling. It is well-known that the estrogen responsive gene BCL2 is a key regulator in the anti-apoptosis process (Samia et al., 2024), so it was further explored whether BCL2 is involved in the apoptosis regulated by FXR1.

A significant positive correlation between BCL2 and FXR1 levels across various cancer types was observed within the TCGA cohort, including breast, prostate, skin cutaneous melanoma, and ovarian cancer (Supplementary Figures S4I–L) (Li et al., 2021). FXR1 depletion led to a marked reduction in both mRNA and protein levels of BCL2 (Figures 4D, E). Moreover, FXR1 depletion increased apoptosis and reduced cell viability were substantially abrogated by forced expression of BCL2 (Figures 4F–J), suggesting FXR1 modulates cell apoptosis via BCL2.

### 3.5 FXR1 promotes BCL2 mRNA maturation

To determine the mechanism utilized by FXR1 to regulate downstream gene expression, RNA immunoprecipitation-sequencing (RIP-seq) was performed to elicit the mRNA-binding landscape of FXR1 in MCF-7 cells (Figure 4K). The aggregate of FXR1 interacting mRNAs was identified by analyzing 618 genes from 2,514 unique peaks (fold change >2 and p < 0.05) (Supplementary Figures S5A). GO analysis revealed the significant enrichment of genes involved in RNA splicing and mRNA metabolism (Supplementary Figures S5B), whereas KEGG analysis linked FXR1 interacting mRNAs with estrogen signaling and endocrine resistance (Supplementary Figures S5C). By defining the RNA binding characteristics of FXR1 in 2,514 peaks, it was found that FXR1 was more inclined to bind to the intronic regions of the identified genes (Supplementary Figures S5D).

Combining RIP-seq data with RNA-seq data, the mRNAs of nine genes were identified to be potentially upregulated and bound by FXR1 (Figures 4L,M). Among these, ESR1 mRNA was reported to bind to FXR1 in a previous study (Yuan et al., 2022). Importantly, BCL2 mRNA, as a participant in FXR1-mediated apoptosis in breast cancer, was also included (Supplementary Figures S5E). After analyzing multiple sites of BCL2 mRNA bound by FXR1, it was observed that the 1081-peak possessed the highest fold change (Figure 4M). Notably, the 1,081 binding site is a GT repeat which is located in the intronic region of the BCL2 gene (Supplementary Figures S5F), implying that FXR1 may bind to BCL2 pre-mRNA. RBPsuite analysis prompted the high affinity interaction of this GT repeat sequence with FXR1 (Supplementary Figure S5G) (Pan et al., 2020). RIP assay was further performed, followed by RT-qPCR to confirm the specific binding of BCL2 pre-mRNA to FXR1 (Figures 4N–P). Interestingly, estrogen significantly boosted the interaction of FXR1 and BCL2 mRNA (Supplementary Figures S5H, I), whereas FXR1 depletion dampened estrogen-induced BCL2 expression (Supplementary Figures S5J, L).

As splicing of introns is necessary for mRNA maturation, it was further determined if FXR1 might affect the maturation of BCL2 mRNA by interaction with its pre-mRNA. RT-qPCR primers were subsequently designed to target the pre-mRNA and mature mRNA of BCL2, respectively. It was shown that FXR1 depletion resulted in the accumulation of BCL2 pre-mRNA (Figures 4Q, R). RIP analysis in nuclear and cytoplasmic fractions showed that FXR1 primarily bound to BCL2 pre-mRNA in the nucleus (Figures 4S–U). This observation was corroborated by a streptavidin pull-down assay using a biotin-labeled GT repeat and His-tagged FXR1 (Figure 4V). Thus, the data suggests that FXR1 promotes BCL2 expression through the regulation of its mRNA maturation.

### 3.6 FXR1 regulates breast cancer ferroptosis

**FIGURE 5:**
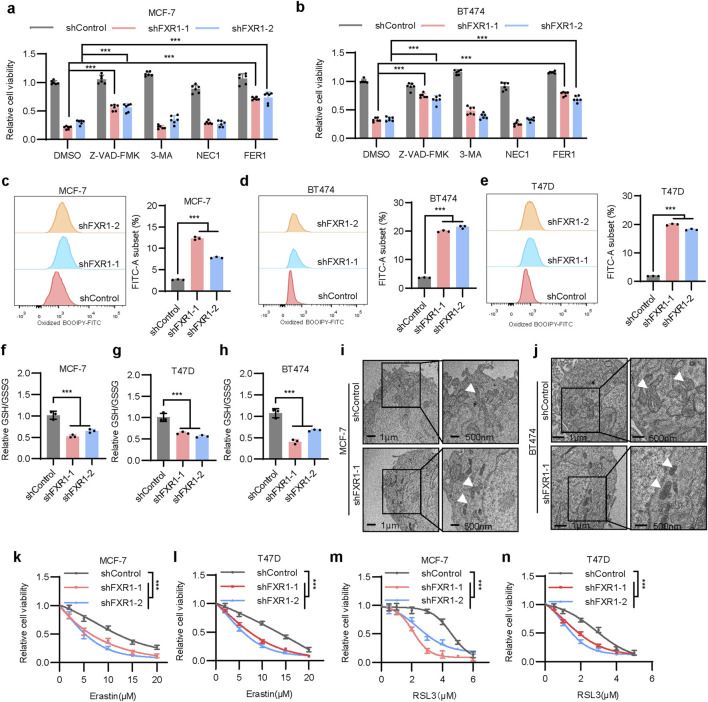
FXR1 depletion promotes ferroptosis. **(a, b)**, Viability of FXR1 depleted MCF-7 **(a)** and BT474 **(b)** cells treated with 5 µM Z-VAD-FMK, 1 mM 3-MA, 1 µM NEC1 or 2 µM FER1 was determined. **(c–e)**, Lipid peroxidation was assessed by flow cytometry after C11-BODIPY staining in FXR1 depleted MCF-7 **(c)**, BT474 **(d)** and T47D **(e)** cells. Representative flow cytometry images (left) and statistical analyses (right) were shown. **(f–h)**, Relative GSH and GSSH levels were measured in FXR1 depleted MCF-7 **(f)**, T47D **(g)** and BT474 **(h)** cells. **(i, j)**, Transmission electron microscopy images show mitochondrial morphology in FXR1 depleted MCF-7 **(i)**, BT474 **(j)**, and control cells. White arrows indicate the mitochondria. **(k–n)**, MTT assays detect the sensitivity of FXR1 depleted MCF-7, T47D, and control cells to erastin or RSL3. Results are shown as mean ± S.D. *P < 0.05; **P < 0.01; ***P < 0.001; ns not significant (Two-way ANOVA test in **(k–n)** others one-way ANOVA test.).

For further characterization of FXR1-regulated cell death, cells were treated with the apoptosis inhibitor Z-VAD-FMK. FXR1 depletion significantly decreased cell viability, which was substantially suppressed by Z-VAD-FMK but not by the autophagy inhibitor 3-MA nor the necroptosis inhibitor NEC1 (Figures 5A, B). Interestingly, a ferroptosis inhibitor, FER1, could partially restore reduced cell viability afforded by FXR1 depletion, suggesting the possible involvement of FXR1 in the regulation of ferroptosis (Figures 5A, B). We subsequently showed that FXR1 depletion in MCF-7, BT474, and T47D cells using shRNA resulted in markedly increased lipid peroxidation, as measured by flow cytometry (Figures 5C–E). Similarly, CRISPR-Cas9 mediated FXR1 deletion enhanced lipid peroxidation in MCF-7 and T47D cells (Supplementary Figures S6A, B). Given that lipid peroxide accumulation is closely linked to the balance between reduced glutathione (GSH) and oxidized glutathione disulfide (GSSG), it was observed that FXR1 depletion significantly reduced the ratio of GSH to GSSG (Figures 5F–H). Transmission electron microscopy revealed that FXR1 depletion caused shrunken mitochondria and increased mitochondrial membrane density, hallmark features of ferroptosis (Figures 5I, J) (Jiang et al., 2021). Consistently, FXR1 depletion increased the sensitivity of breast cancer cells to ferroptosis inducer erastin or RSL3 (Figures 5K–N). It is interesting to note that no apparently altered ferroptosis or apoptosis was observed in ER- MDA-MB-231 cells following FXR1 depletion (Supplementary Figures S6C, D). The above data indicate that FXR1 plays important roles in regulating apoptosis and ferroptosis, particularly in ER+ breast cancer.

### 3.7 FXR1 regulates ferroptosis via GPX4

**FIGURE 6:**
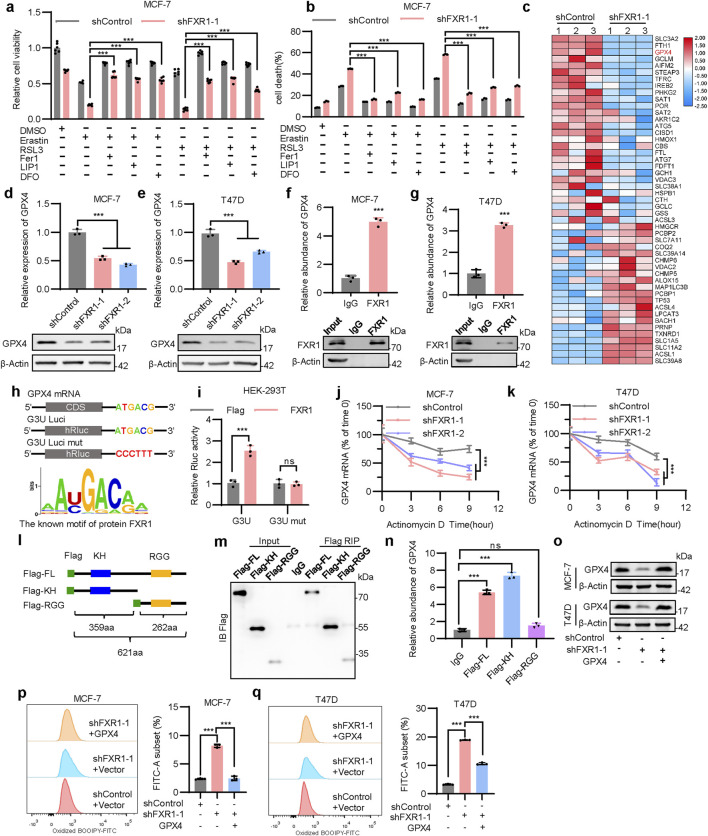
FXR1 interacts with the GPX4 mRNA to regulate ferroptosis. **(a, b)**, Viability **(a)** and death **(b)** of FXR1 depleted MCF-7 cells were detected, treated with 10 µM erastin or 1 µM RSL3 combined with 2 µM FER1, 1 µM LIP1 or 5 µM DFO. **(c)**, Heat map analysis of RNA-seq data showing mRNA expression of ferroptosis-related genes in FXR1 depleted cells compared to control cells. **(d, e)**, qPCR (n = 3 biological replicates) and immunoblot analysis of GPX4 expression in FXR1 depleted MCF-7 **(d)** and T47D **(e)** cells. **(f, g)**, qPCR (n = 3 biological replicates) analyzed the interaction of FXR1 with GPX4 mRNA by RIP assays in MCF-7 **(f)** and T47D **(g)** cells. **(h)**, Schematic representation of luciferase reporter plasmids containing full-length and mutated GPX4-3′UTR (up). FXR1 recognition motif predicted by RBPsuite was shown (down). **(i)**, Luciferase reporter plasmids were co-transfected with FXR1 expression plasmid or vector control in HEK-293T cells, and luciferase activities were determined. **(j, k)**, FXR1 depleted MCF-7 **(j)** and T47D **(k)** cells were treated with actinomycin **(d)**. GPX4 mRNA were examined at the indicated time points. **(l)**, Schematic diagram of full-length and domain mutated of FXR1. **(m, n)**, Flag-FL, Flag-KH, or Flag-RGG were transfected in HEK-293T cells for RIP assays using Flag antibody. Immunoblot **(m)** and qPCR (n = 3 biological replicates) **(n)** were performed to analyze the association of different FXR1 domains with GPX4 mRNA. **(o)**, Immunoblot assessment of GPX4 levels in FXR1-depletion MCF-7 and T47D cells rescued with empty vector or GPX4 plasmid. **(p, q)**, Lipid peroxidation was assessed in FXR1 depleted MCF-7 **(p)** and T47D **(q)** cells rescued with empty vector or GPX4 plasmid. Results are shown as mean ± S.D. *P < 0.05; **P < 0.01; ***P < 0.001; ns not significant (Unpaired two-tailed Student’s t test in **(f, g, i)**, two-way ANOVA test in **(j, k)** others one-way ANOVA test.).

It was observed that FXR1 depletion afforded inhibition of cell viability could be significantly reversed by ferroptosis inhibitors, including FER1, LIP1, and DFO (Figure 6A; Supplementary Figure S6E). Consistently, the reversion of cell death was confirmed by flow cytometry analysis in FXR1-depleted MCF-7 and T47D cells (Figure 6B; Supplementary Figure S6F). To gain mechanistic insight into FXR1 regulated ferroptosis in breast cancer, the expression profile of ferroptosis-related genes (https://www.wikipathways.org/pathways/WP4313) was examined using RNA-seq data generated from FXR1 depleted cells (Figure 4A). It was shown that multiple ferroptosis related genes were downregulated, including SLC3A2, FTH1, and GPX4, as a result of FXR1 depletion (Figure 6C). As GPX4 plays a pivotal role in the ferroptosis related antioxidant system (Chen et al., 2024), the regulatory mechanism of FXR1 on GPX4 was explored. Consistent with the RNA-seq data, FXR1 depletion using shRNA led to significantly reduced mRNA and protein levels of GPX4 (Figures 6D,E). It was further confirmed that the protein level of GPX4 was also significantly decreased in cells with deletion of FXR1 (Supplementary Figure S6G). The above data imply that depletion of FXR1 promotes ferroptosis by down-regulating the expression of GPX4.

The possible interaction between FXR1 and the 3′UTR of GPX4 was subsequently examined. RIP assay demonstrated that GPX4 mRNA was indeed enriched in FXR1 precipitates (Figures 6F, G). Analysis using RBPsuite identified a highly conserved FXR1 recognition motif within GPX4 mRNA 3′UTR (Figure 6H). To determine if FXR1 influences GPX4 expression by binding to its 3′UTR, two luciferase reporters were constructed: G3U-Luci containing the full-length 3′UTR of GPX4, and G3U-Luci mut containing the 3′UTR of GPX4 with the FXR1 recognition motif mutated (Figure 6H). It was shown that forced expression of FXR1 markedly increased the luciferase activity of G3U-Luci, whereas the activity of G3U-Luci mut remained unaltered (Figure 6I), suggesting that FXR1 regulates GPX4 expression via binding to its 3′UTR. Further analysis revealed that FXR1 depletion significantly reduced the half-life of GPX4 mRNA (Figures 6J, K). As FXR1 has been reported to regulate gene expression via its KH and RGG domains (Edwards and Joseph, 2022), flag fusion proteins containing various domains (Flag-FL, Flag-KH, Flag-RGG) of FXR1 were generated for RNA immunoprecipitation analysis (Figure 6L). It was observed that GPX4 mRNA binds to the Flag-FL and Flag-KH fusion proteins but not Flag-RGG in HEK-293T cells (Figures 6M, N), highlighting the critical role of the KH domain of FXR1 in its interaction to GPX4 mRNA. It was further demonstrated that GPX4 depletion in MCF-7 cells led to decreased viability and increased lipid peroxidation (Supplementary Figures S6H–J). To verify that FXR1 depletion promoted ferroptosis is dependent on GPX4, the expression of GPX4 was forced in cells lacking FXR1 (Figure 6O). The results showed that forced expression of GPX4 significantly restored the accumulation of lipid peroxidation caused by FXR1 depletion (Figures 6P, Q). Furthermore, the decreased cell viability due to FXR1 depletion was significantly restored by forced expression of GPX4 in the presence of erastin (Supplementary Figures S6K, L). Overall, FXR1 promotes the expression of GPX4 by binding to GPX4 mRNA, leading to suppressed ferroptosis.

### 3.8 FXR1 promotes anti-estrogen resistance in ER+ breast cancer

As FXR1 is regulated by estrogen, whether FXR1 affects the estrogen responsiveness of ER+ breast cancer cells was investigated. Estrogen deprived MCF-7 and T47D cells were treated with 10 nM estrogen. It was observed that FXR1 depletion reduced estrogen-promoted foci formation and cell viability (Supplementary Figures S7A–D), indicating a key role of FXR1 in mediating estrogen elicited functionality in ER+ breast cancer cells. As the response to estrogen is closely linked to the sensitivity to anti-estrogen in breast cancer treatment (Ozyurt and Ozpolat, 2022), it was further investigated whether estrogen regulated FXR1 is involved in endocrine resistance. Two cell models with endocrine resistance were established, namely, cells with acquired resistance due to long-term exposure to tamoxifen (TAMR) and the cells exposed to long-term estrogen deficiency (LTED) (Mansouri et al., 2017).

**FIGURE 7:**
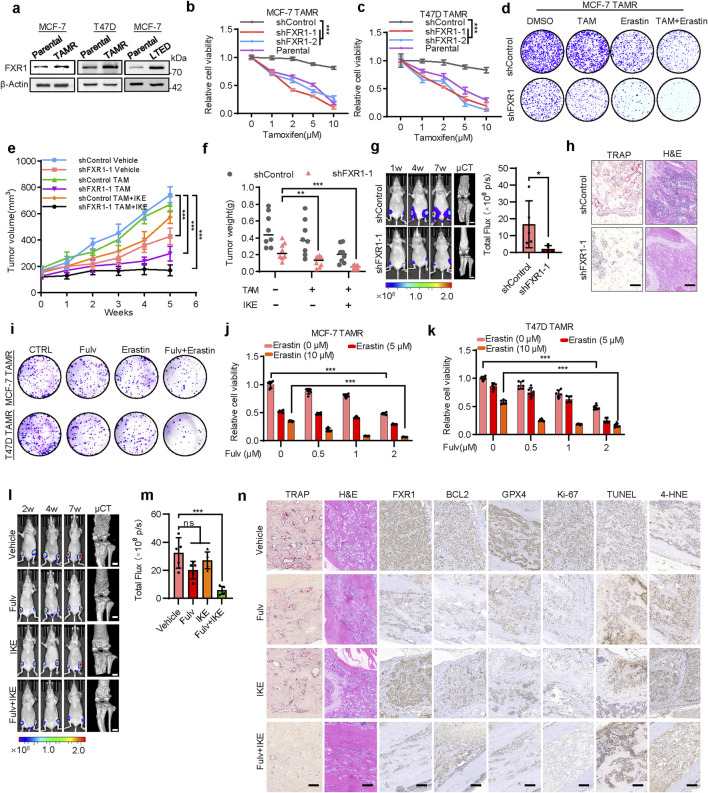
FXR1 promotes anti-estrogen resistance and bone metastasis. **(a)**, Immunoblot assessment of FXR1 levels in TAMR, LTED and cognate parental cells. **(b, c)**, The sensitivity of FXR1 depleted MCF-7 TAMR **(b)** and T47D TAMR **(c)** cells to tamoxifen was evaluated by MTT assays. **(d)**, FXR1 depleted MCF-7 TAMR cells were treated with 5 µM tamoxifen, 10 µM erastin, or both, and foci formation assays were performed. **(e, f)**, MCF-7 TAMR cells stably expressing pLKO1 or sh-FXR1 vector were injected into nude mice (n = 8/group). After the tumor size reached 150 mm^3^, the mice were treated with tamoxifen and IKE. Tumor growth curves **(e)** and tumor weight **(f)** are shown. **(g)**, Representative images of bioluminescence (BLI) and micro-CT of bone metastasis through caudal artery injection of 1.5 × 10^6^ MCF-7 TAMR-shControl or -shFXR1 cells into nude mice (n = 5/group). BLI quantification of bone metastases in nude mice is shown on the right. **(h)**, Representative immunohistochemical images of TRAP and H&E staining were taken in each group. Scale bars: 50 μm. **(i)**, Foci formation assay was performed by using FXR1 depleted MCF-7 TAMR and T47D TAMR cells treated with 1 µM fulvestrant, 10 µM erastin, or both. **(j, k)**, Cell viability was measured by MTT assay by using FXR1 depleted MCF-7 TAMR **(j)** and T47D TAMR cells **(k)** treated with fulvestrant and erastin. **(l)**, Representative images of BLI and micro-CT of bone metastasis through caudal artery injection of 2 × 10^6^ MCF-7 TAMR cells into nude mice. The mice were treated with vehicle, fulvestrant, IKE, or fulvestrant + IKE. **(m)**, BLI quantification of bone metastases in nude mice from **(l)**. **(n)**, Representative TRAP, H&E, and immunostaining images of FXR1, BCL2, GPX4, Ki67, TUNEL, and 4-HNE were shown from sections of **(l)**. Scale bars: 50 µm. Results are shown as mean ± S.D. *P < 0.05; **P < 0.01; ***P < 0.001; ns not significant (Unpaired two-tailed Student’s t test in **(g)**, two-way ANOVA test in **(b, c and e)** others one-way ANOVA test.).

The maintenance of tamoxifen insensitivity of the existing TAMR model was verified using foci formation assay (Supplementary Figures S7E, F) (Yuan et al., 2022). LTED cells were cultured for at least 6 months in phenol red-free RPMI medium supplemented with 5% dextran charcoal-stripped bovine serum. The LTED cells exhibited hypersensitivity to low concentrations of estrogen (such as 10^−12^ M) and increased lethality upon high concentrations of estrogen (such as 10^−9^ M) (Supplementary Figures S7G, H) (Song et al., 2001; Martin et al., 2003; Martin et al., 2005). Consistent with the previous analysis using the GSE164529 dataset (Figure 1C), FXR1 levels were significantly increased in MCF7-TAMR, T47D-TAMR, and LTED cells compared to their cognate controls (Figure 7A). The capacity of FXR1 in regulating cell viability (Supplementary Figures S7I–K) as well as apoptosis and ferroptosis in anti-estrogen resistant cell lines was revealed (Supplementary Figures S7L–Q), aligning with its functionality in cognate parental cells. Importantly, FXR1 depletion restored the sensitivity of TAMR cells to tamoxifen (Figures 7B, C). Consistently, FXR1 depletion increased whereas forced expression of FXR1 reduced the sensitivity of parental cells to tamoxifen (Supplementary Figures S8A–D). These results suggested that FXR1 antagonism might be used for enhancing the efficacy of anti-estrogen therapy.

As ferroptosis induction is increasingly recognized as a promising strategy for cancer treatment (Li et al., 2020), the possibility of combining this strategy in the anti-estrogen therapy of breast cancer was further examined. It was observed that combined treatment of TAMR cells with tamoxifen and erastin significantly restored their sensitivity to tamoxifen (Figure 7D; Supplementary Figures S8E–G). FXR1 depletion further enhanced this synergy, resulting in a much enhanced sensitivity to tamoxifen (Figure 7D; Supplementary Figures S8E–G). Consistently, FXR1 depletion led to significantly elevated cell death when treated with tamoxifen and erastin compared to the controls (Supplementary Figures S8H, I). Further investigation of the efficacy of combinatorial treatment was performed *in vivo* using TAMR cells. It was observed that IKE (an erastin analog) treatment significantly restored the sensitivity of the TAMR cells derived xenografts to tamoxifen (Figure 7E, F; Supplementary Figures S8J). The xenografts derived from FXR1-depleted cells also regained sensitivity to tamoxifen compared to the controls (Figures 7E, F). Remarkably, the combinatorial treatment with tamoxifen and IKE exerted significantly enhanced efficacy in abrogating the growth of xenografts derived from FXR1-depleted cells compared to the controls (Figures 7E, F; Supplementary Figure S8J). IHC results confirmed reduced expression of BCL2, GPX4, and Ki-67, along with increased levels of TUNEL and 4-HNE in FXR1-depleted cancer cells compared to the controls (Supplementary Figure S8K).

### 3.9 Fulvestrant combined with IKE inhibits bone metastasis of endocrine resistance breast cancer

Patients with long-term anti-estrogen therapy often suffer from lethal recurrence and distant metastases, especially bone metastases (Liang et al., 2020). To investigate whether FXR1 was involved in regulating bone metastasis of ER+ breast cancer, luciferase-labeled TAMR cells were injected into nude mice through the intra-caudal arterial, a murine model of bone metastasis (Kuchimaru et al., 2018). Bioluminescent imaging analyses showed that the xenografts derived from FXR1-deleted TAMR cells exhibited much reduced growth compared to the controls throughout the development of bone metastasis (Figure 7G). Micro-computed tomography imaging (μCT) also revealed much decreased bone lesions in the FXR1-silenced group compared to the control group (Figure 7G). Tartrate-resistant acid phosphatase (TRAP) staining confirmed the reduced number of activated osteoclasts in the shFXR1 group (Figure 7H). Consistently, H&E staining showed significantly fewer lesions in the bone tissue of shFXR1 group compared to the control group (Figure 7H). IHC analysis showed that bone metastases derived from FXR1 depleted TAMR cells exhibited reduced expression of BCL2, GPX4, and Ki-67. Furthermore, TUNEL and 4-HNE levels increased in bone metastases derived from FXR1 depleted TAMR cells compared with the control group (Supplementary Figure S8L). These results suggest that FXR1 antagonism might be used for suppressing bone metastasis derived from ER+ breast cancer cells.

Fulvestrant has been proven effective in treating anti-estrogen resistant ER+ breast cancer by degrading ERα as the second line of treatment (Hutcheson et al., 2003). Considering that fulvestrant can inhibit the expression of FXR1, the feasibility of combining fulvestrant with IKE to treat bone metastasis was also explored. The combined treatment of fulvestrant with erastin significantly inhibited the foci formation ability and cell viability of TAMR cells *in vitro* (Figures 7I–K). By using the TAMR cell derived bone metastasis model, it was observed that the combinatorial therapy effectively abrogated xenograft growth as well as bone damage compared to single-agent treatment (Figures 7L, M). There was no significant change of mouse weight with either single agent or combinatorial treatment, suggesting the tolerance of the combined therapy (Supplementary Figure S8M). TRAP and H&E staining confirmed that the combined treatment significantly inhibited the extent of bone damage (Figure 7N). Histological analysis indicated that the combination of fulvestrant and IKE significantly reduced FXR1, BCL2, and GPX4 expression in bone metastases, whereas elevated apoptosis and ferroptosis levels were reflected by TUNEL and 4-HNE staining, respectively (Figure 7N). Therefore, the combination of anti-estrogen therapy and inducers of ferroptosis may present a promising therapeutic strategy for breast cancer patients with bone metastasis due to failed primary endocrine therapy.

## 4 Discussion

Dysregulated estrogen signaling is a critical factor contributing to anti-estrogen resistance and bone metastasis in breast cancer (Saatci et al., 2021; Mohammadi Ghahhari et al., 2022). Since there is no superior option to replace anti-estrogen therapy at present, there is a critical need to develop novel approaches to overcome anti-estrogen resistance in ER+ breast cancer. Herein, the screening of ERα regulated mRNA-binding proteins by iTRAQ technology combined with public dataset analysis identified FXR1 as a novel ERα-regulated oncogenic gene. The expression of FXR1 was further subjected to translational regulation by eIF4E via estrogen and IGF-1 stimulated PI3K/AKT/mTOR pathway. The PI3K/AKT/mTOR pathway is commonly aberrant in both primary and recurrent ER+ breast cancer, leading to acquired endocrine resistance (Nunnery and Mayer, 2020; Dong et al., 2021). As the crosstalk between the ER and RTK signaling pathways play a critical role in the development of ER+ breast cancer (Lee et al., 2022), the study herein presented a paradigm utilized by these signaling pathways to converge on FXR1 for propelling anti-estrogen resistance. FXR1 may thus serve as an interesting biomarker for monitoring the efficacy of anti-estrogen treatment and possible development of acquired endocrine resistance.

Recent studies have shown that the escape of cancer cells from both apoptosis and ferroptosis are associated with treatment failure in cancer patients (Szostakowska et al., 2019; Li et al., 2020). Herein, it was demonstrated that FXR1 plays a critical role in the development of anti-estrogen resistance by directly interacting with the key regulators of apoptosis and ferroptosis, BCL2 and GPX4, respectively. FXR1 has been reported to interact with various other RNAs, such as p21, TERC, and c-MYC, exerting regulatory effects in different cancer types (Majumder et al., 2016; Majumder et al., 2020; George et al., 2021). Herein, it is reported that FXR1 binds to BCL2 pre-mRNA to promote its maturation and expression; and FXR1 increases GPX4 expression by stabilizing its mRNA via interaction with the 3′UTR. Thus, FXR1 utilizes both anti-apoptosis and anti-ferroptosis signaling to promote cancer progression. It is fascinating to note that the FXR1 exerted effects on apoptosis and ferroptosis tend to be ER+ breast cancer cell specific, indicating a critical role of ER signaling in conditioning the functionality of FXR1, consistent with the observation that the association of poor prognosis with FXR1 expression was exclusively associated with ER+ but not ER- breast cancer patients. It is expected that estrogen promotion of FXR1 expression combined with the abundance of BCL2 and GPX4 in ER+ breast cancer (Sha et al., 2021; Kawiak and Kostecka, 2022) should facilitate FXR1 to impinge on both apoptosis and ferroptosis signaling. Interestingly, estrogen also significantly enhanced the interaction of FXR1 and BCL2 mRNA. As FXR1 was reported to be regulated by different post-translational modifications (PTMs), which affect its capacity to interact with different RNAs(Vijayakumar et al., 2024), whether estrogen or IGF-1 signaling should modulate FXR1 by PTMs would be interesting to determine in future studies.

Herein, it was shown that FXR1 expression was elevated in ER+ breast cancer and further increased in anti-estrogen resistant breast cancer cells. As FXR1 acts downstream of ERα and IGF1R signaling, it might serve as an attractive therapeutic target in ER+ breast cancer with deregulated ERα activity. Consistently, targeting FXR1 by shRNA not only significantly increased the efficacy of tamoxifen in ER+ breast cancer cells, but restored the sensitivity to tamoxifen in tamoxifen-resistant cells *in vivo*. FXR1 antagonism combined with ferroptosis inducer IKE further increased the efficacy of tamoxifen therapy. Proof-of-principle evidence was provided that FXR1 antagonism could alleviate bone metastasis of ER+ breast cancer cells with acquired anti-estrogen resistance, wherein PI3K/AKT/mTOR signaling is often abnormally activated (Song et al., 2022). As suggested by the efficacy of this approach, combined therapy with fulvestrant and IKE successfully targeted both apoptosis and ferroptosis signaling in the bone metastasis model. Thus, targeting FXR1 may afford a novel approach to improve existing therapeutic regimes for ER+ breast cancer patients with recurrent cancer or bone metastasis with acquired resistance to anti-estrogen therapies.

## Data Availability

The datasets presented in this study can be found in online repositories. The names of the repository/repositories and accession number(s) can be found in the article/Supplementary Material.
