# Supplementary material for: Estrogen-induced FXR1 promotes endocrine resistance and bone metastasis in breast cancer via BCL2 and GPX4
Source: Front Cell Dev Biol. 2025 Mar 24;13:1563353. doi: 10.3389/fcell.2025.1563353 (PMC11973456; doi:10.3389/fcell.2025.1563353)
Supplement: Supplementary file 3 [file DataSheet1.docx]

Supplementary Material

## Supplementary Figures


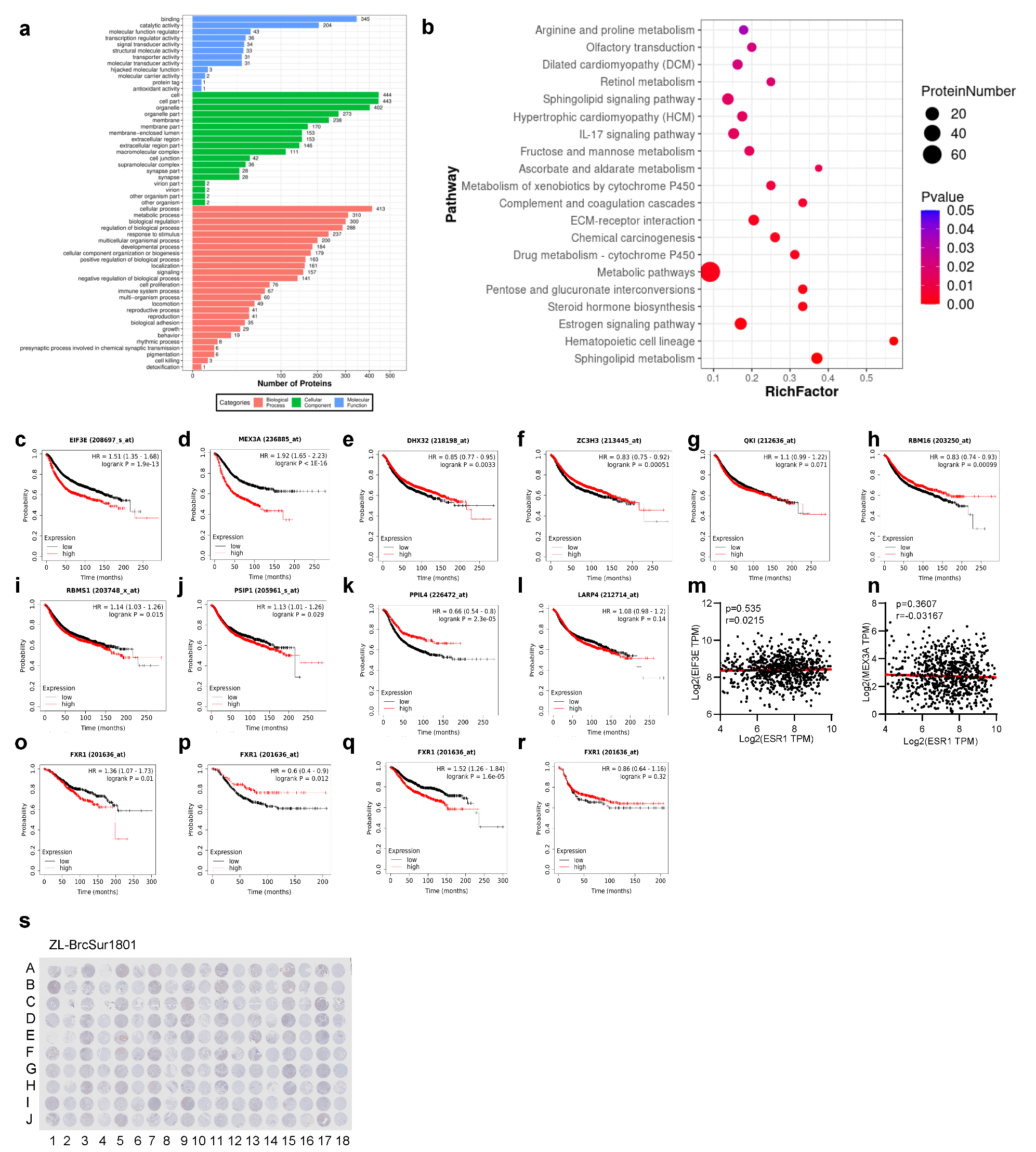


**Supplementary Fig.1 Estrogen-related FXR1 is associated with poor prognosis of ER+ breast cancer, related to Figure 1. a**, Gene ontology analysis of differentially expressed proteins in iTRAQ. **b**, Statistics of pathway enrichment of differentially expressed proteins in iTRAQ. **c-l**, Kaplan–Meier plots of RFS in breast cancer patients with different levels of EIF3E (**c**), MEX3A (**d**), DHX32 (**e**), ZC3H3 (**f**), QKI (**g**), RBM16 (**h**), RBMS1 (**i**), PSIP1 (**j**), PPIL4 (**k**) and LARP4 (**l**). **m**, **n**, Scatter plot represents the correlation between ESR1 and either EIF3E (**m**) or MEX3A (**n**). **o**, **p**, Kaplan–Meier plots of OS in ER+ (**o**) and ER- (**p**) patients with different levels of FXR1 expression. **q**, **r**, Kaplan–Meier plots of DMFS in ER+ (**q**) and ER- (**r**) patients with different levels of FXR1 expression. **s**, IHC staining of FXR1 using breast cancer tissue microarray (tumor tissues (1,3,5…17), normal tissues (2,4,6…18)).

**
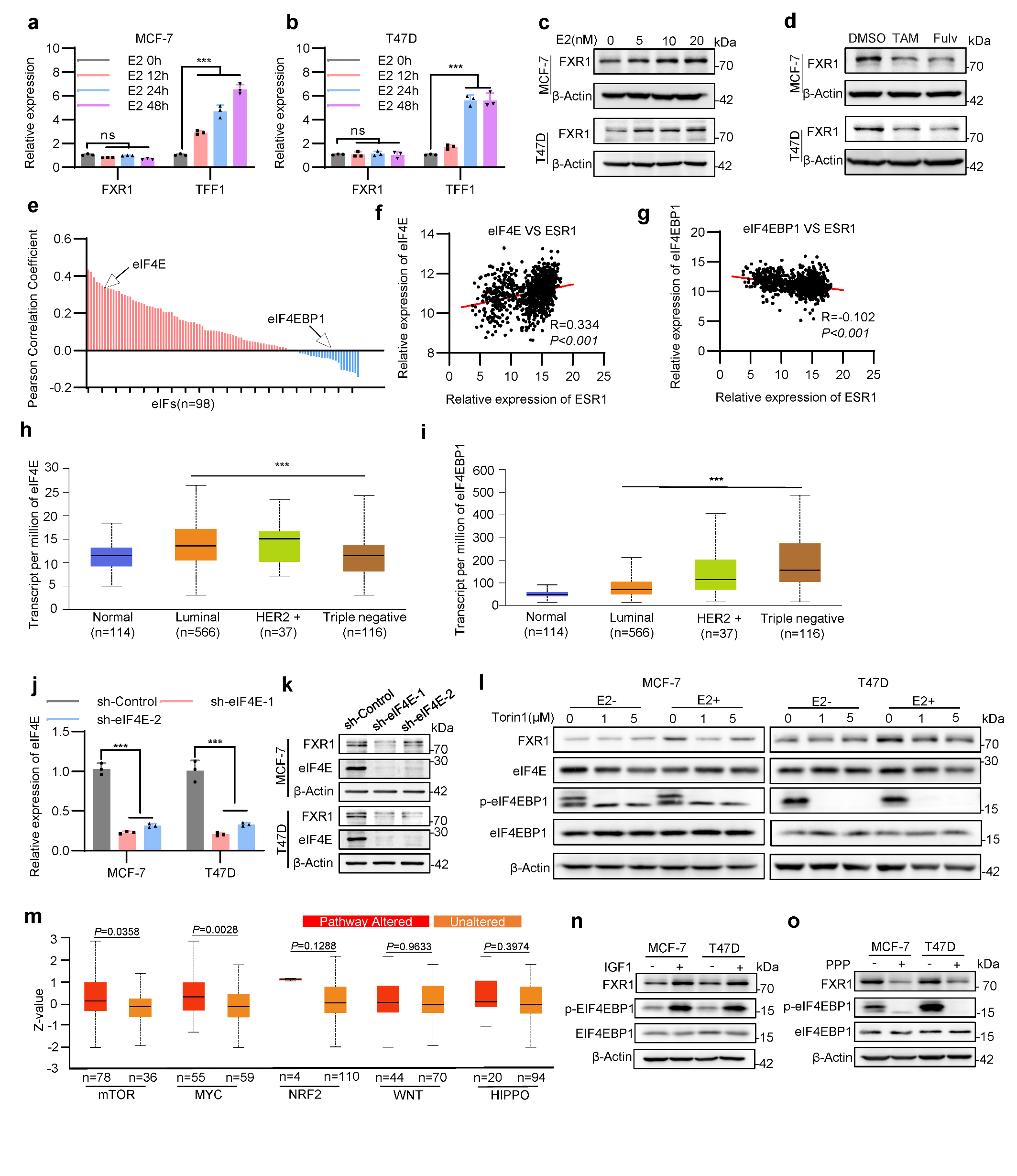
**

**Supplementary Fig.2 Estrogen induces FXR1 translation through eIF4E and eIF4EBP1, related to Figure 2. a**, **b**, qPCR (n = 3 biological replicates) analysis of relative mRNA levels of FXR1 and TFF1 in MCF-7 (**a**) and T47D (**b**) cells treated with 10 nM estrogen for 0h, 12h, 24h, and 48h. **c**, **d**, Immunoblot assessment of FXR levels in cells treated with different concentrations of estrogen (**c**), 1 μM tamoxifen, or 1 μM fulvestrant (**d**). **e**, Correlation analysis of eukaryotic translation initiation factors with ESR1 in the TCGA dataset. **f**, **g**, Analysis of the Pearson correlation coefficient (PCC) between ESR1 and eIF4E (**f**) or eIF4EBP1 (**g**) in the TCGA dataset. **h**, **i**, Expression of eIF4E (**h**) and eIF4EBP1 (**i**) in major subclasses of breast cancer was analyzed by UALCAN. **j**, **k**, qPCR (n = 3 biological replicates) (**j**) and immunoblot (**k**) analysis of eIF4E levels in eIF4E depleted MCF-7, T47D and control cells. **l**, Immunoblot assessment of FXR1, eIF4E and phosphorylated eIF4EBP1 levels. MCF-7 and T47D cells were treated with different concentrations of Torin1 (mTOR inhibitor) in the presence or absence of estrogen. **m**, FXR1 protein expression levels in breast cancer tissues with different altered signaling pathways. **n**, **o**, Immunoblot assessment of FXR1, eIF4EBP1 and phosphorylated eIF4EBP1 levels. MCF-7 and T47D cells treated with 50 ng/mL IGF1 (**n**) or 1 μM PPP (**o**). Results are shown as mean ± S.D. *P < 0.05; **P < 0.01; ***P < 0.001; ns not significant (One-way ANOVA test).

**
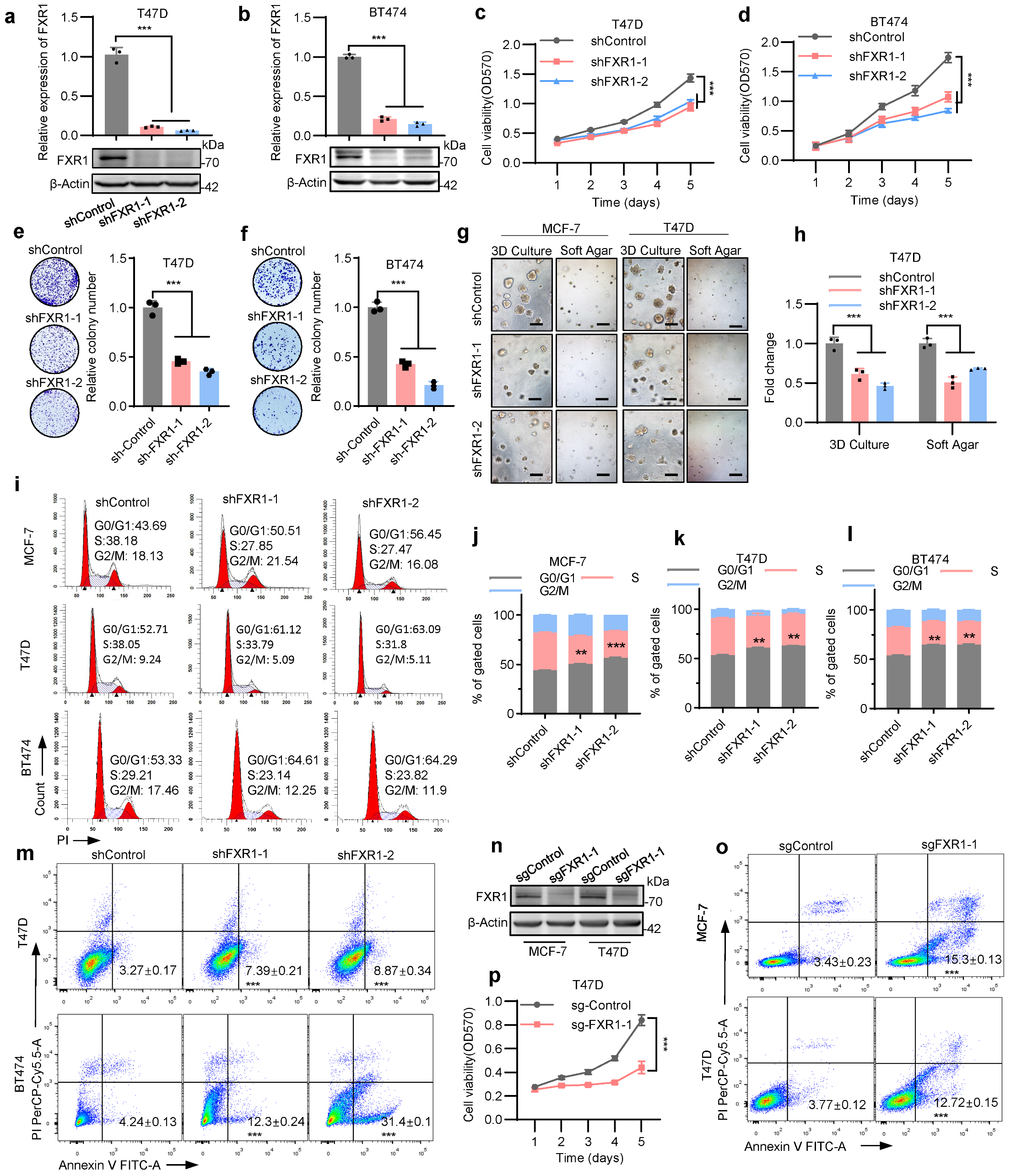
**

**Supplementary Fig. 3 FXR1 promotes the oncogenicity of breast cancer cells, related to Figure 3. a**, **b**, qPCR (n = 3 biological replicates) and immunoblot analysis of FXR1 expression in FXR1 depleted T47D (**a**), BT474 (**b**) and control cells. **c**, **d**, MTT assay showing relative cell viability in FXR1 depleted T47D (**c**), BT474 (**d**) and control cells. **e**, **f**, Foci formation assay was performed in FXR1 depleted T47D (**e**), BT474 (**f**) and control cells. Representative images (left) and statistical analyses (right) of the colonies were shown. **g**, **h**, 3D culture and soft agar colony formation assays were performed in FXR1 depleted and control cells. Representative images (**g**) and statistical analyses (**h**) of the colonies were shown (scale bars:3D culture 100 μm, soft agar culture 500 μm). **i**, Flow cytometry showing the cell cycle distribution of FXR1 depleted and control cells. **j-l**, Statistical analyses of cell cycle phases of FXR1 depleted MCF-7 (**j**), T47D (**k**), and BT474 (**l**) cells by flow cytometry. **m**, Early apoptotic population in FXR1 depleted T47D, BT474 and control cells was determined by flow cytometry. **n**, Immunoblot assessment of FXR1 levels in FXR1-deleted MCF-7, T47D and control cells. **o**, Early apoptotic population in FXR1-deleted MCF-7, T47D and control cells was determined by flow cytometry. **p**, MTT assay showing relative cell viability in FXR1-deleted T47D and control cells. Results are shown as mean ± S.D. *P < 0.05; **P < 0.01; ***P < 0.001; ns not significant (Unpaired two-tailed Student’s t test in (**o**), two-way ANOVA test in (**c**, **d**, **p**) others one-way ANOVA test.)

**
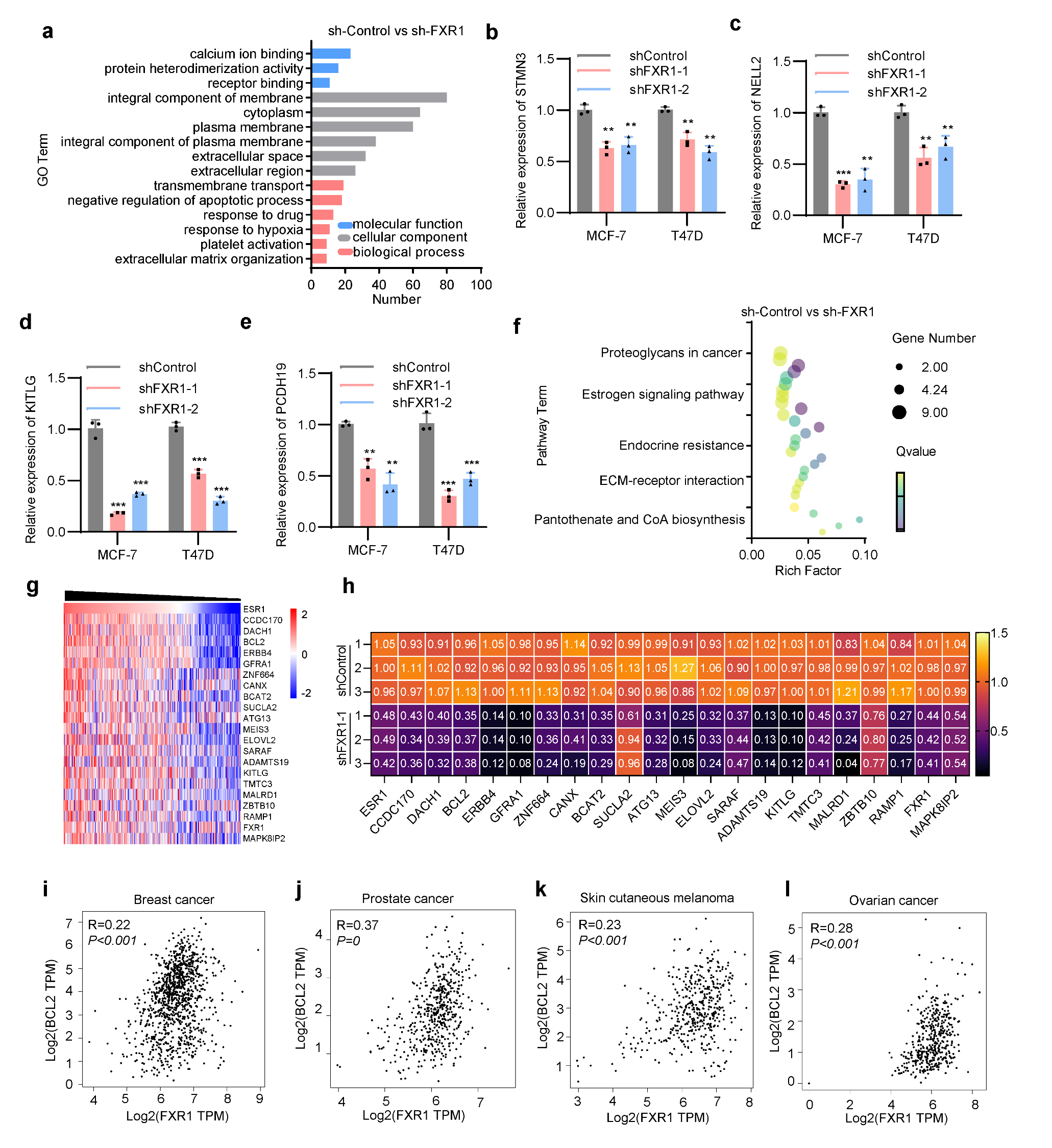
**

**Supplementary Fig. 4 FXR1 protects cells against apoptosis by regulating BCL2 expression, related to Figure 4. a**, Gene ontology analysis of differentially expressed genes in RNA-seq. **b-e**, qPCR (n = 3 biological replicates) analyzed expression of STMN3 (**b**), NELL2 (**c**), KITLG (**d**), and PCDH19 (**e**) in FXR1 depleted MCF-7, T47D and control cells. **f**, KEGG pathway analysis of differentially expressed genes in RNA-seq. **g**, Heat map plots the relative expression levels of candidate genes and ESR1 in breast cancer TCGA data. **h**, qPCR (n = 3 biological replicates) analysis of candidate genes expression in FXR1 depleted MCF-7 and control cells. **i-l**, Correlation analysis of FXR1 and BCL2 in breast (**i**), prostate (**j**), skin cutaneous melanoma (**k**), and ovarian cancer (**l**) TCGA cohorts using GEPIA2. Results are shown as mean ± S.D. *P < 0.05; **P < 0.01; ***P < 0.001; ns not significant (One-way ANOVA test.)


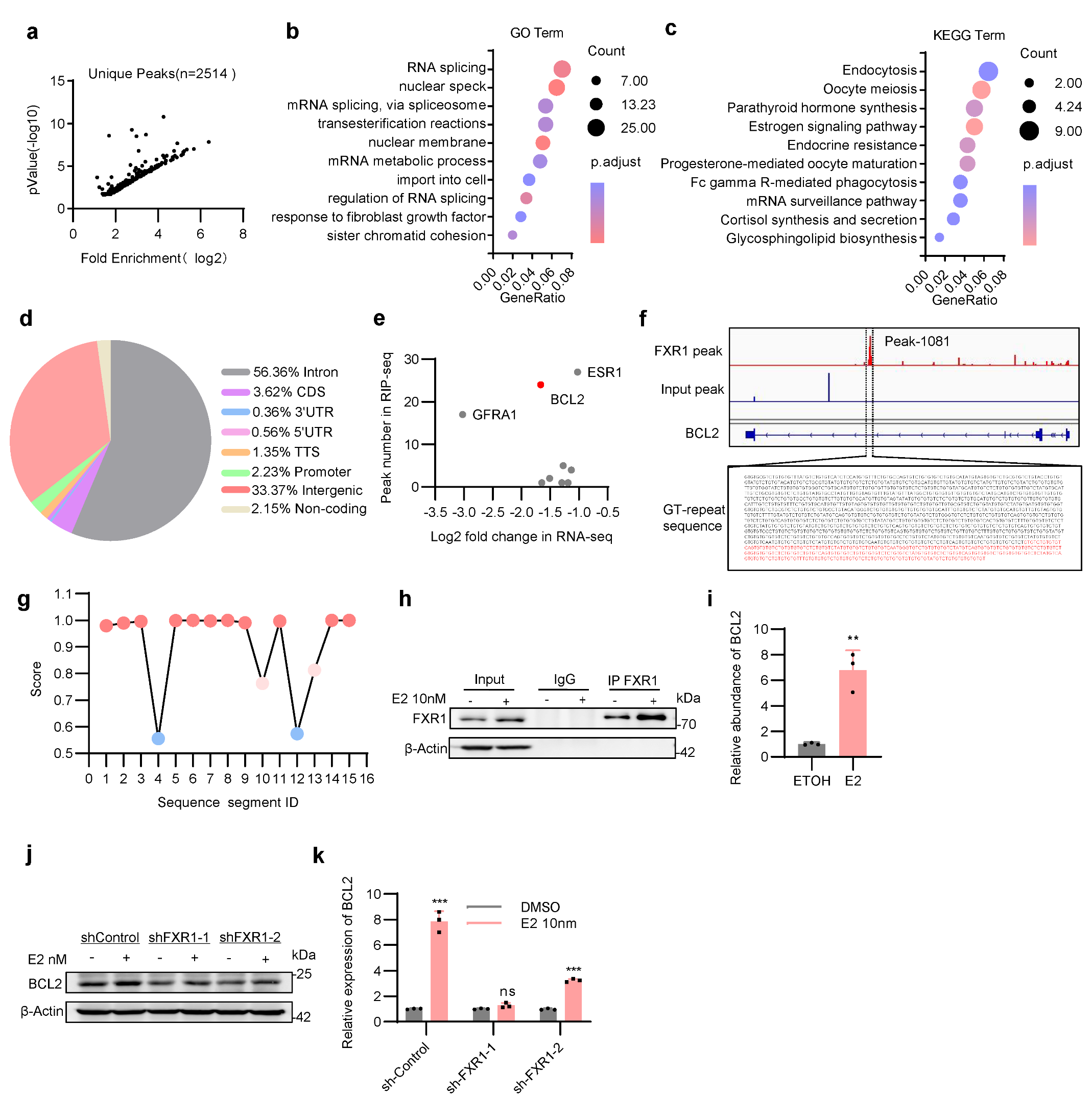


**Supplementary Fig. 5 FXR1 regulates BCL2 mRNA maturation, related to Figure 4. a,** Scatterplot showing 2514 unique peaks enriched by FXR1 in RIP experiments. **b**, **c**, Gene ontology (**b**) and KEGG pathway (**c**) analysis of 618 genes directly bound by FXR1 in RIP experiments. 10 representative terms were shown for each analysis. **d**, Pie chart showing the distribution of 2514 unique peaks in functional regions across the genome. **e**, Scatterplot showing the number of unique peaks in the RIP experiment and the fold change in RNA-seq of the screened 9 genes. Negative values of fold change ​​represent downregulation in FXR1 depleted cells. **f**, Visualization of unique peaks bound by FXR1 on the BCL2 genome using IGV software (up). Below is the entire DNA sequence of Peak-1081, and the red sequence is labeled with biotin for the streptomycin pull-down experiment. **g**, The binding ability of FXR1 to the mRNA sequence of Peak-1081 was analyzed using RBPsuite. **h-i,** MCF-7 cells were stimulated with vehicle or estrogen for 48 hours, and RIP experiments were performed using FXR1 antibody. The enrichment level of FXR1 was detected by immunoblot (**h**). qPCR (n = 3 biological replicates) (**i**) analysis of the BCL2 mRNA enrichment level in RIP experiments. **j**, **k,** Immunoblot (**j**) and qPCR (n = 3 biological replicates) (**k**) analysis of BCL2 expression levels in vehicle or estrogen stimulated FXR1 depleted MCF-7 and control cells. Results are shown as mean ± S.D. *P < 0.05; **P < 0.01; ***P < 0.001; ns not significant (Unpaired two-tailed Student’s t test)


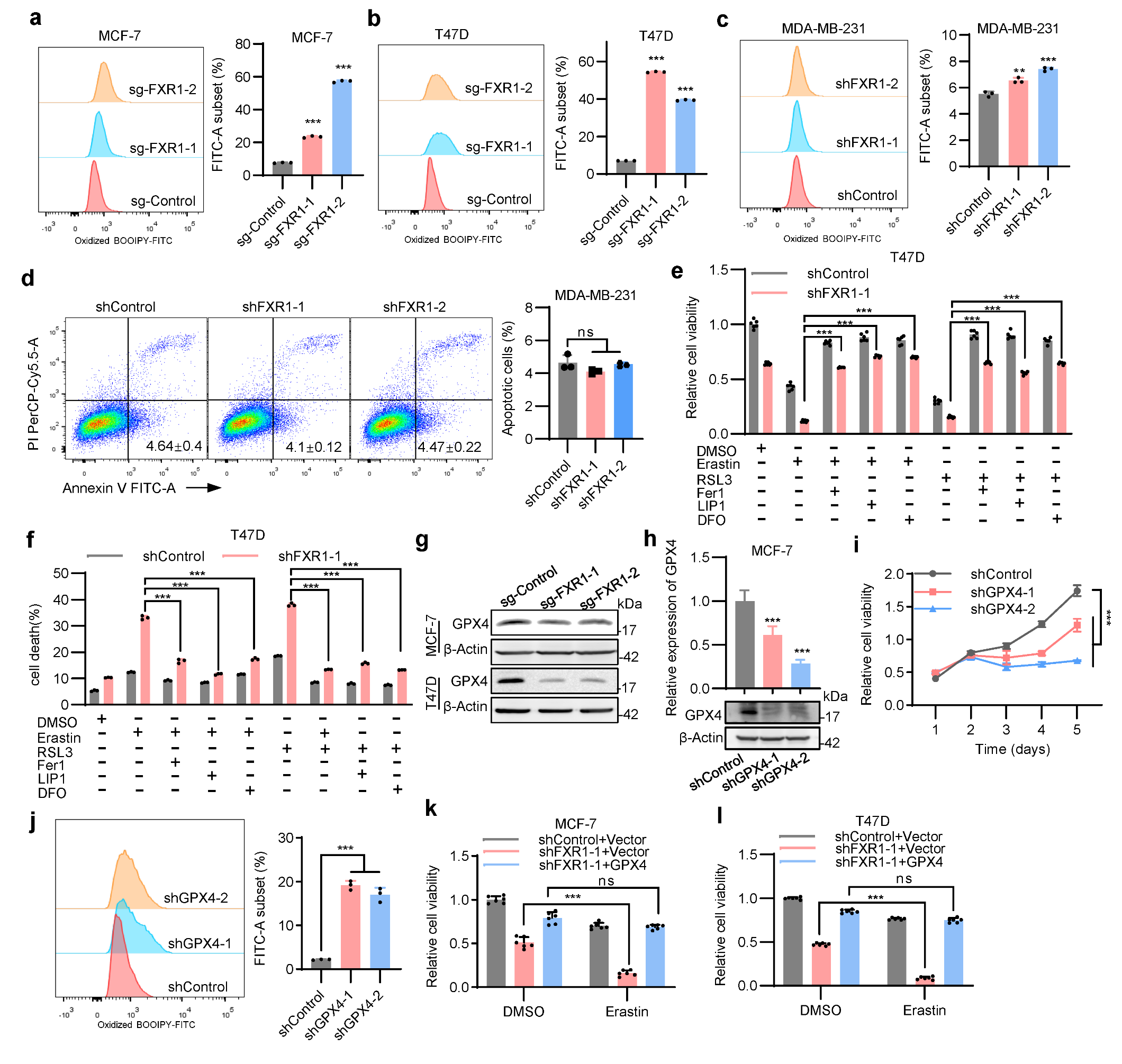


**Supplementary Fig. 6 FXR1 protects breast cancer cells from ferroptosis via GPX4, related to Figure 6. a-c**, Lipid peroxidation was assessed by flow cytometry after C11-BODIPY staining in FXR1-deleted MCF-7 (**a**), T47D (**b**) and FXR1-depleted MDA-MB-231 cells (**c**). Representative flow cytometry images (left) and statistical analyses (right) were shown. **d**, Early apoptotic population in FXR1 depleted MDA-MB-231 and control cells was determined by flow cytometry. Representative flow cytometry images (left) and statistical analyses (right) were shown. **e**, **f**, Viability (**e**) and death (**f**) of FXR1 depleted T47D and control cells were detected, treated with 10 µM erastin or 1 µM RSL3 combined with 2 µM FER1, 1 µM LIP1 or 5 µM DFO. **g**, Immunoblot assessment of GPX4 levels in FXR1-deleted MCF-7, T47D and control cells. **h**, qPCR (n = 3 biological replicates) and immunoblot analysis of GPX4 expression in GPX4 depleted MCF-7 and control cells. **i**, Viability of GPX4 depleted MCF-7 and control cells were detected by MTT assay. **j**, Lipid peroxidation was assessed by flow cytometry after C11-BODIPY staining in GPX4 depleted MCF-7 cells. Representative flow cytometry images (left) and statistical analyses (right) were shown. **k**, **l**, FXR1 depleted MCF-7 (**k**) and T47D (**l**) cells were rescued with empty vector or transfection of the GPX4 plasmid and treated with DMSO or erastin. Subsequently, cell viability was determined by MTT assay. Results are shown as mean ± S.D. *P < 0.05; **P < 0.01; ***P < 0.001; ns not significant (Two-way ANOVA test in (**i**) others one-way ANOVA test.)


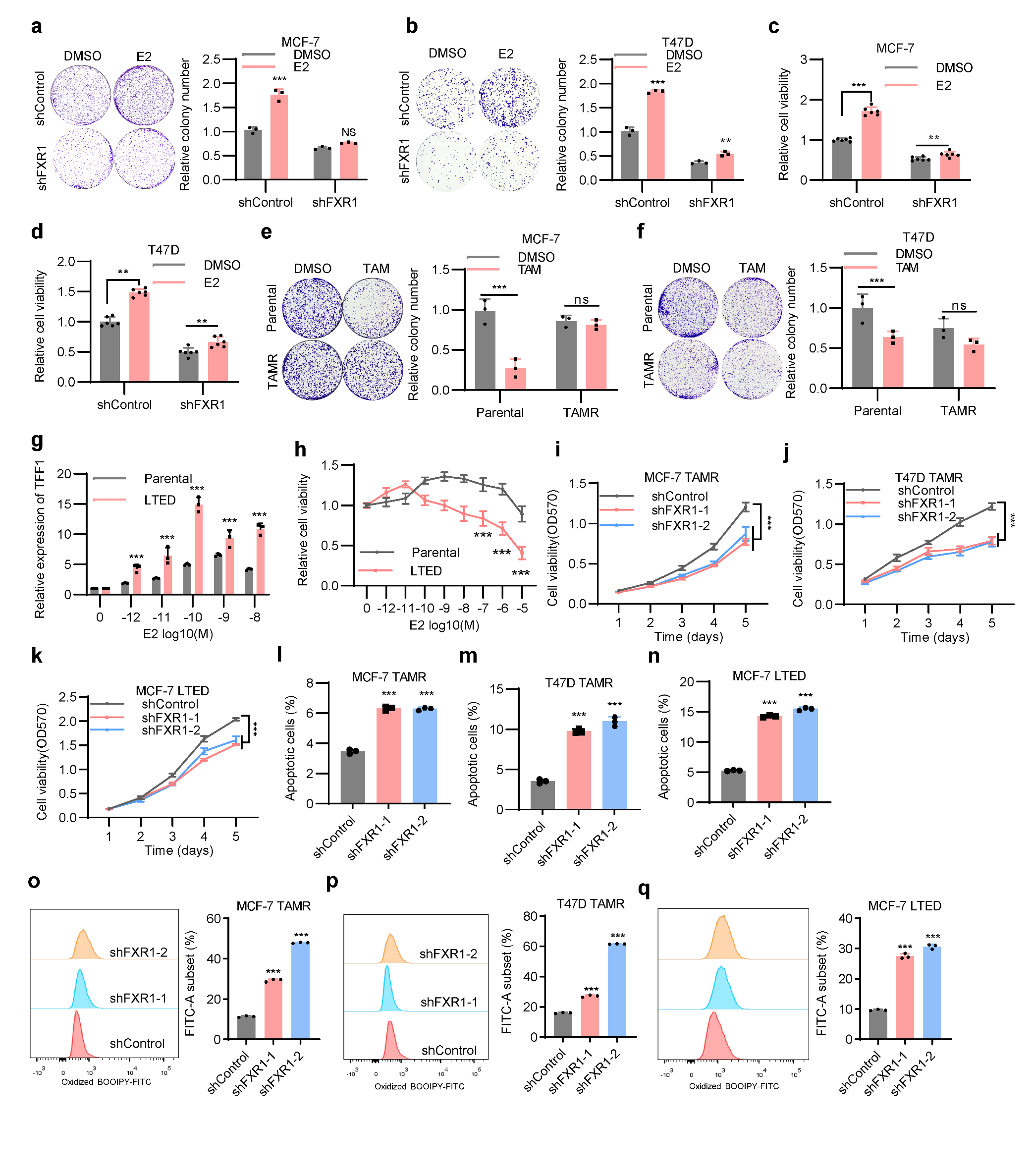


**Supplementary Fig. 7 Depletion of FXR1 leads to apoptosis, ferroptosis, and re-sensitization to tamoxifen in TAMR cells, related to Figure 7. a-d**, FXR1 depleted MCF-7, T47D and control cells were treated with 10 nM estrogen, foci formation (**a**, **b**) and MTT assays (**c**, **d**) were performed. **e**, **f**, Foci formation assays were performed in MCF-7-TAMR, T47D-TAMR, and parental cells treated with 5 μM tamoxifen. **g**, **h**, MCF-7 parental and LTED cells treated with indicated concentrations of estrogen for 48 hours. qPCR (n = 3 biological replicates) analysis of TFF1 expression (**g**). The cell viability was detected by MTT assay (**h**). **i-k**, MTT assay showing cell viability in FXR1 depleted MCF-7-TAMR (**i**), T47D-TAMR (**j**), and MCF-7 LTED (**k**) cells within 5 days. **l-n**, Early apoptotic population in FXR1 depleted MCF-7-TAMR (**l**), T47D-TAMR (**m**), MCF-7 LTED (**n**) and control cells was determined by flow cytometry. **o-q**, Lipid peroxidation of FXR1 depleted MCF-7-TAMR (**o**), T47D-TAMR (**p**), MCF-7 LTED (**q**) and control cells were determined by flow cytometry. Results are shown as mean ± S.D. *P < 0.05; **P < 0.01; ***P < 0.001; ns not significant (Two-way ANOVA test in (**i**, **j**, **k**), one-way ANOVA test in (**l-q**), others unpaired two-tailed Student’s t test.)


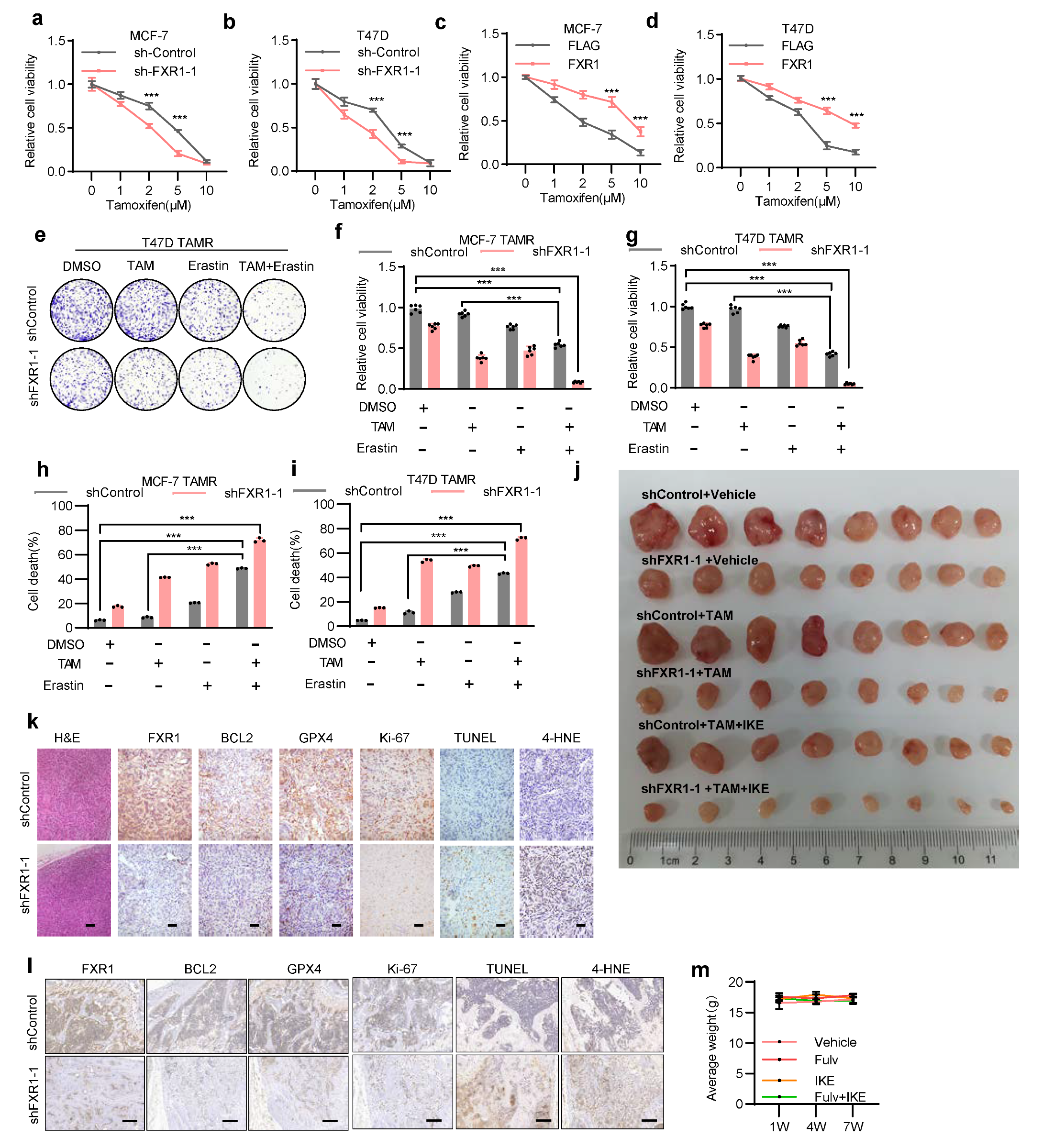


**Supplementary Fig. 8 Combined treatment with tamoxifen and IKE for breast cancer xenografts,** **related to Figure 7. a**-**d**, MTT assays assessing the sensitivity of MCF-7 and T47D cells with FXR1 depleted (**a**, **b**) or forced expressed (**c**, **d**) to tamoxifen. **e**, FXR1 depleted T47D TAMR and control cells were treated with 5 µM tamoxifen, 10 µM erastin, or both, and foci formation assay was performed. **f**, **g**, FXR1 depleted MCF-7 TAMR (**f**), T47D TAMR (**g**) and control cells were treated with 5 µM tamoxifen, 10 µM erastin, or both, and MTT assay was performed. **h**, **i**, FXR1 depleted MCF-7 TAMR (**h**), T47D TAMR (**i**) and control cells were treated with 5 µM tamoxifen, 10 µM erastin, or both. The proportion of dead cells was determined by flow cytometry. **j**, Tumor pictures of Figure 7e. **k**, Representative H&E and IHC images of FXR1, BCL2 GPX4, Ki67, TUNEL, and 4-HNE in the experiment of Figure 7e. Scale bar: 100 μm (H&E), 50 μm (IHC). **l**, Representative immunostaining images of FXR1, BCL2, GPX4, Ki67, TUNEL, and 4-HNE in the experiment of Figure 7g. Scale bars: 50 µm. **m**, Body weight curve of mice in the experiment of Figure 7l. Results are shown as mean ± S.D. *P < 0.05; **P < 0.01; ***P < 0.001; ns not significant (Unpaired two-tailed Student’s t test.)
